# Group music therapy with songwriting for adult patients with long-term depression (SYNCHRONY study): a feasibility and acceptability study of the intervention and parallel randomised controlled trial design with wait-list control and nested process evaluation

**DOI:** 10.1186/s40814-023-01285-3

**Published:** 2023-05-05

**Authors:** Catherine Elizabeth Carr, Emma Millard, Merve Dilgul, Cornelia Bent, Donald Wetherick, Jennifer French, Stefan Priebe

**Affiliations:** 1 Unit for Social and Community Psychiatry, WHO Collaborating Centre for Mental Health Services Development, Centre for Psychiatry and Mental Health, Wolfson Institute of Population Health, Queen Mary University of London, Newham Centre for Mental Health, Glen Road, London, E13 8SP UK; 2 East London NHS Foundation Trust, Trust Headquarters, Robert Dolan House, 9 Alie Street, London, E1 8DE UK

**Keywords:** Chronic depression, Long-term depression, Group music therapy, Songwriting, Randomised controlled trial, Feasibility

## Abstract

**Background:**

Despite effective treatments, one fifth of patients develop chronic depression. Music therapy may offer a different approach. This study aimed to assess feasibility and acceptability of a music therapy intervention and trial methodology.

**Methods:**

A parallel two-arm randomised controlled trial with wait-list control, mixed feasibility/acceptability measures and nested process evaluation. Adults with long-term depression (symptom duration > 1 year) were recruited from community mental health services and computer randomised to 42 sessions of group music therapy with songwriting three times per week or wait-list control. Depression, social functioning, distress, quality of life, satisfaction and service use were assessed by blinded researchers at enrolment, 1 week and 3 and 6 months post-therapy. Outcomes were analysed descriptively, controlling for baseline covariates. Recruitment (number eligible, participation and retention rates) and intervention (fidelity, adherence) feasibility were assessed using pre-defined stop–go criteria. Attendance, adverse events, mood, relationship satisfaction and semi-structured interviews were analysed in a nested process evaluation.

**Results:**

Recruitment processes were feasible with 421 eligible, 12.7% participation and 60% (18/30) retention. Thirty participants were randomised to intervention (*N* = 20) and control (*N* = 10). Session attendance was low (mean 10.5) with four withdrawals. Music therapist adherence was good but changes to session frequency were suggested. Outcomes were available for 10/20 treatment and 9/10 wait-list participants. Depression increased in both arms post-therapy. Treatment depression scores fell below baseline 3 and 6 months post-therapy indicating improvement. Wait-list depression scores increased from baseline 3 and 6 months post-therapy. At 3 months, the treatment arm improved from baseline on all measures except satisfaction and functioning. At 6 months, quality of life, distress and functioning improved with reduction in health service contacts. High-attending participants improved more than low-attending. Seven adverse events (one serious) were reported.

**Limitations:**

As this was a feasibility study, clinical outcomes should be interpreted cautiously.

**Conclusion:**

A randomised controlled trial of group music therapy using songwriting is feasible with inclusion criteria and session frequency modifications, but further intervention development is required.

**Trial registration:**

ISRCTN18164037 on 26.09.2016.

**Supplementary Information:**

The online version contains supplementary material available at 10.1186/s40814-023-01285-3.

## Key messages regarding feasibility

- What uncertainties existed regarding the feasibility?

Music therapy is a promising intervention for depression but has not been tested in a group songwriting format for long-term depression. We were also uncertain about the numbers that would meet our definition of long-term depression and how best to identify and recruit them to our study.

- What are the key feasibility findings?

The study methods were feasible and acceptable to participants and we were able to recruit sufficient numbers within the timeframe required. Group attendance was low, with a high proportion not attending a single session, and initial high attrition. Inclusion criteria may require a more stringent assessment of depression severity and this may aid identification of participants more likely to attend the intervention. Outcomes suggested a worsening of symptoms post-intervention in both arms before improvements 3 months later. The intervention requires further modification in terms of frequency, location, music therapist technological support and support for group members once the groups come to an end.

- What are the implications of the feasibility findings for the design of the main study?

Recruitment is most successful from secondary mental health services, with options for patient self-referrals. Further development of the intervention and piloting to determine the primary endpoint are required before a larger trial is implemented.

## Background

The global burden of depression is well-recognised: Despite many effective treatments, around one in five diagnosed with an acute depressive disorder develops chronic depression [1]. The severity and course of symptoms vary from ‘milder’ symptoms of dysthymia to chronic major depression [2]. For this specific patient group, median durations are estimated between 5 and 20 years [3, 4] with associated increased health care costs through greater use of services and rates of hospitalisation [5–7]. Known risk factors include younger age of onset, childhood adversity and abuse [8–18], family history of mood disorder and problems within the social environment (such as low social integration, support and negative social interaction) [3].

Chronic or persistent depression is defined by symptoms lasting 2 or more years. However, durations of 1 year or longer are still both clinically relevant (in terms of distress) and may be indicative of a chronic course [8, 19]. Around 40% of chronically depressed patients fulfil the criteria for treatment resistance, which can be identified as soon as 6 months post-diagnosis (or after two trials of antidepressant drugs) [20]. This suggests that symptoms enduring for 1 year or longer are both an indicator of future chronicity and a need for further intervention. For the purposes of this study, we use the term ‘long-term depression’ to define patients with symptoms of depression that have lasted 1 year or longer.

Treatment of long-term depression is particularly difficult: Frequent relapses can lead to pessimism and demoralisation of both patient and professional [4] leading, in turn, to a lack of compliance or ‘giving up’ on treatment. There is evidence for both pharmacotherapy [21, 22] and psychotherapy [23] as effective treatments. These effects appear to be maximised when used in combination [24] although around 18 sessions of psychotherapy may be necessary in order to see clinical effects [25]. A later review found limited evidence for their use in combination [26] but suggested psychotherapy might have a continued role in promoting and maintaining treatment adherence, given patient preferences are often for psychotherapy over medication and achieving wider clinical benefits (such as improved coping strategies and quality of life). As a result, clinical guidelines recommend combined treatment with a personalised approach [9].

There is good evidence for psychotherapy interventions that target interpersonal problems (such as the cognitive behavioural analysis system of psychotherapy (CBASP) and interpersonal psychotherapy (IPT) [27]. Similarly, long-term psychoanalytic psychotherapy has been shown to improve long-term outcomes in treatment-resistant depression [28]. Given the social environment is a known risk factor for this population [3, 29], group formats may promote social integration and interaction, provide emotional and social support and offer potential cost-effectiveness.

## Group music therapy

Music therapy is a complex intervention provided by music therapists that uses a range of expressive and receptive musical activities, verbal reflection and the relationships developed through this to improve health [30]. Within the United Kingdom (UK), music therapists are regulated by the Health and Care Professions Council (HCPC) and must have completed accredited Masters level training. Within the UK, practice most often uses a combination of active musical improvisation and verbal reflection within sessions, which can take an individual or group format.

There is promising evidence for the effectiveness of music therapy in treating depression [31] and it may benefit this population for several reasons. As an intervention, it may be appealing and motivating given the different focus on use of the art form and thus encourage attendance and engagement [32, 33]. The experience of making music provides a very different therapeutic encounter; music has an immediate impact (often positive) on mood [34] and within groups (especially singing) can promote social bonding [35]. A positive experience within a community-based group may then place the person in contact with their musical and psychological ‘resources’ [36], which—linking to wider theories of recovery in mental illness—may provide opportunities to build inner resources of coping, resilience and promote hope [37, 38].

Through co-created musical improvisation, it is possible to give sound to experience, express and transform feeling states, form relationships and communicate with others without words. These experiences may promote opportunities for more positive social interactions than those experienced verbally. The musical attunement facilitated by music therapists when improvising may help patients to experience nonverbal social contact, closeness, emotional containment and address feelings of social isolation [39]. This process is implicated in building initial therapeutic trust, which is an important factor for this patient group [40]. Notably, a randomised controlled trial of individual psychodynamic improvisational music therapy for depression [41] found additional benefits on alexithemia, suggesting that musical improvisation assisted patients in naming internal feeling states.

A further music therapy trial used group songwriting for patients with severe mental illness and demonstrated improved quality of life [33]. Creating bespoke songs as a group has the potential for participants to begin to find ways of putting their internal experiences into words and to have this supported through group discussion and music-making [42].

Clinical benefits are associated with the number of sessions received. One meta-analysis [43] suggested around 4 sessions would be required for a small effect on depressive symptoms, 10 for a medium effect and 16 for a large effect. The impact of session frequency and duration is less clear. Within the UK, sessions are often offered on a weekly basis. However, internationally, frequency can range from 1 to 6 sessions per week [43].

In designing the intervention for this study, we consulted with patient and carer groups, who suggested that singing would be a more accessible and acceptable way of making music than instrumental improvisation. They also emphasised the importance of having an ‘end product’ in promoting self-esteem, self-efficacy and achievement in their recovery. We therefore took a group songwriting protocol [33] as our starting point and through focus groups with music therapists and clinical psychologists and interviews with patients with depression, incorporated principles from psychodynamic improvisational music therapy [40] and resource-oriented music therapy [36, 44].

By offering a regular intensive group format (3 sessions per week), we hypothesised that patients would have opportunities to make music together thus providing opportunities to build trust and bond with others, improve mood and build relationships. We hypothesised this could lead to a range of relevant outcomes such as short-term reduction in psychological distress and improved social functioning. The above could also contribute to improved self-esteem and self-efficacy and, taken as a whole, a reduction in depression symptoms. Secondary impacts of a reduction in depression were hypothesised to be improved satisfaction with services, a reduced impact of depression upon work and life and improved quality of life.

Current evidence suggests group music therapy may offer an alternative and potentially clinically beneficial treatment for long-term depression. However, the intervention has not been specified or tested specifically for this population using a group and songwriting format within a UK National Health Service (NHS). Whilst music therapy is commonly provided in NHS mental health care, provision is often to diagnostically heterogeneous groups. Similarly, whilst songwriting is a recognised music therapy technique, it is less frequently used in the UK. It was therefore important to assess whether the intervention was delivered as described, and its general acceptability to both patients and music therapists.

In terms of the research design, it was important to assess our proposed methods for identifying, recruiting and retaining participants. In particular, we were unsure of the numbers who might meet our definition of ‘long-term’ depression, where they might be identified within services, nor of the best ways to identify them. Running the study on a small scale enabled us to examine how feasible our proposed processes were and to estimate the resources and most effective approaches required [45]. We were similarly unsure which measures might be most appropriate in terms of acceptability of completion, the variability of outcomes and what level of clustering might be expected within groups.

## Aims and objectives

This study aimed to pilot a group songwriting music therapy intervention for patients with long-term depression and assess the feasibility and acceptability both of the intervention and of conducting a larger randomised controlled trial. In addition, the study sought to gather descriptive information on health service use in order to inform a future health economic evaluation.

### Objectives

- Feasibility and acceptability of research methodology
  1. Assess the feasibility of recruitment processes
  2. Identify the number of eligible participants, participation and retention rates
  3. Assess the researcher time required
  4. Assess the appropriateness of outcome measures, including providing data on the variability of outcome, an estimate of the control group mean and the intra-cluster correlation coefficient.
  5. Assess the acceptability of the research methodology to professionals and patients
- Feasibility and acceptability of intervention
  6. Assess the intervention in terms of testing use of components, measuring adherence and estimating the likely intervention effect.
- Assessment of service use for health economic evaluation
  7. Assess the services received by participants in preparation for a health economic evaluation.

## Methods

A parallel two-arm randomised controlled feasibility trial with mixed methods evaluation. Participants were assessed at the point of enrolment (baseline), the week post-intervention and 3 and 6 months post-intervention. Shopping vouchers of £10 were offered at baseline and for subsequent assessments for treatment participants. Wait-list participants were paid £15 per follow-up to acknowledge the delay to treatment. The study was given favourable ethical opinion from the Health Research Authority (IRAS project ID: 198,964, REC reference:16/WA/0248), and the study protocol was published with open access in March 2017 [46].

Four amendments were made during the study. We amended the patient information sheet and consent form to include the possibility of payment for travel to therapy sessions where patients did not hold a ‘freedom pass’; a substantial amendment was made to move the post-test assessment point from 1 month post-intervention to immediately at the intervention end to maximise follow-up rates and capture any immediate treatment effects; we clarified payment of £10 for participation in qualitative interviews to ensure consistency with previous assessments; finally, prior to commencing music therapy for the wait-list group, we opened up two spaces to patients outside the study to ensure a critical mass of group members could be maintained.

### Eligibility criteria

As this was a feasibility trial, our inclusion criteria were as broad as possible. Participants were eligible if they had a confirmed diagnosis in the International Classification of Diseases and Related Health Problems (version 10) (ICD10), of depression (ICD10 F31-39), including post-schizophrenic depression (ICD10 F20.4) and prolonged depressive reaction (ICD10 F43.21), had received pharmacological and/or psychological treatment for 12 months or longer, were aged 18 years or above and had capacity to give written informed consent. We excluded any diagnosis of organic mental disorder (ICD10 F00-09), bipolar affective disorder if current manic episode (ICD10 F30, F31.0, F31.2, F31.6, F31.7–4), if they lacked capacity to give informed consent or were at risk of suicide necessitating hospitalisation. Previous receipt of music therapy or other psychological therapies did not form part of the eligibility criteria, but were recorded as part of baseline clinical characteristics.

### Setting and participant identification

The study took place in East London NHS Foundation Trust. Research assistants recruited participants via (a) primary care, via General Practice (GP) surgeries and (b) secondary care via improving access to psychological therapies (IAPT) services and community mental health care teams. GP surgeries were invited to sign up to act as recruiting centres. A practice staff member then sent letters of invitation to any potentially eligible patients. Within secondary care, caseloads were screened by a clinical studies officer who was part of the care team and potential participants were approached by the professional responsible for their care. An unexpected third means of recruitment was via patient self-referral through presentations about the study to patient and carer groups across the Trust. Where patients expressed interest, permission was gained to contact their healthcare professional to check eligibility and then a meeting arranged to go through informed consent.

### Participant consent

Recruitment lasted for 8 weeks between September and November 2016. Interested patients were provided with an information sheet and then met with a member of the research team to give written informed consent and complete baseline measures. To support retention, we aimed wherever possible for the researcher conducting baseline assessments to continue with that participant for all follow-up assessments.

### Intervention (Group music therapy with songwriting)

The Synchrony group music therapy with songwriting intervention is summarised according to the Template for Intervention Description and Replication (TIDieR) checklist [47] in Table 1. A manual for the Synchrony group music therapy with songwriting intervention [Additional file 1], based on Grocke et al. [33] and informed by individual psychodynamic music therapy for depression [40] and resource-oriented music therapy [36], was developed prior to the study taking place through focus groups with music therapists, psychologists and interviews with patients with depression. The manual was finalised through regular meetings with the music therapists providing the intervention and Heads of Arts Therapies.

**Table 1:** TIDieR [47] summary of the Synchrony group music therapy with songwriting intervention

TIDieR item	Description
1 Brief name	Synchrony group music therapy with songwriting for chronic depression
2 Why	Chronic depression is associated with challenges with low mood, motivation and social isolation. Group formats may promote social integration, interaction, provide emotional and social support and offer potential cost-effectiveness [3, 29] Music therapy has promising evidence in treating depression [31] and offers a different therapeutic encounter. The intervention may be appealing and motivating encouraging attendance and engagement. Music has an immediate (often positive) impact upon mood [34] which may reduce symptom distress and within groups (especially singing), can promote social bonding [35]. Musical improvisation may support initial nonverbal communication of feeling states and aid patients in learning to name these [41]. Group songwriting may further aid verbal expression of internal experiences and is associated with improved quality of life [33]. Patient and carer groups value the accessibility of singing and importance of an ‘end product’ in promoting self-esteem, self-efficacy and achievement in recovery By offering a regular intensive group format, patients will have opportunities to make music together thus providing opportunities to build trust and bond with others, improve mood and build relationships. We hypothesise this will lead to short-term reduction in psychological distress and improved social functioning. The above will contribute to improved self-esteem and self-efficacy and taken as a whole, a reduction in depression symptoms. Secondary impacts of reduced depression will be improved satisfaction with services, reduced impact of depression upon work and life and improved quality of life
3 What: materials	• Range of large and hand held percussion instruments, e.g. large: Djembe drum, bongos, conga, snare, tom toms / small: cabassa, castanets, cowbell, triangle, various shakers, chimes
	• Tuned instruments: guitar, electric keyboard and/or acoustic piano, auto harp, xylophone, ballaphone, marimba, glockenspiel, harmonica, thumb piano, chime bars, hand bells, etc
	• 2–4 microphones for recording and stand
	• Recording equipment: zoom digital audio recorder, iPad with compatible external microphone and Garageband or similar software
	• Amplification for iPad and electric guitar/keyboard where required
	• Projector to connect to iPad for song ideas
	• Speakers for playback
	• Flipchart and blu-tack
4 What: procedures	Group music therapy with songwriting, based on an adapted songwriting intervention [33] and informed by psychodynamic music therapy for depression [40] and resource-oriented music therapy [36]
	1. Pre-therapy induction session with music therapists to meet each other, set expectations, answer questions and introduce the equipment and sorts of music-making that will happen
	2. Text message reminders sent to participants to encourage group attendance
	3. First session: Extended introductions, overview of 14-week schedule, group rules, introduction to songwriting
	4. General group structure and format: Instrumental/body warm up and check in. Initial sessions use reflection on a piece of music brought to the session by a group member. Music therapists encourage group discussion. Warm-up improvisation using a theme from previous discussion to prepare for songwriting. Group reflection on the experience and ideas/themes they wish to take forward into the songwriting. Group songwriting with option to rehearse and/or perform elements. End of session check in on how feeling now compared to the beginning. Reflection on the group events and decisions
	5. Sessions 2–31: Songwriting and developing group song list
	6. Sessions 32–42: Group review and closure—Sessions are dedicated to reviewing the songs written, including possibility to rehearse and record or perform. Reflection on group processes and relationships
5 Provider	Two HCPC-registered NHS music therapists
6 How	Face-to-face, group format, up to 10 participants per group
7 Where	Community centre, room with space to seat up to 12 (10 participants and 2 music therapists). Some décor such as paintings, plants, natural light. Reasonable soundproofing from interior to exterior. Room to be free from interruption or loud external noise for duration of session. Wifi to enable access to the internet for song-sharing and mobile phone signal
8 When/how much	
a) Intensity	High intensity
b) Frequency	Three sessions per week
c) Session time	90 min consisting of 60 min session with 15 min pre/post for socialisation
d) Overall duration	14 weeks
9 Tailoring	Group structure was permitted to become more flexible (in terms of improvisation and songwriting content) as sessions progressed to tailor to the evolving needs of the group. Songwriting elements are used interchangeably where appropriate to aid the songwriting process (creating lyrics, developing the song, choosing genre, developing rhythmic structure, developing verse/chorus melody, choosing mode/harmony, adding instrumental accompaniment/possibilities for improvisation, rehearsing, final song performance)
10 Modifications	Participants unable to attend regularly were encouraged and supported to stay in contact with the music therapists and to return when they could. This meant some participants attended only once or twice per week, and some did not attend for an extended period in the group therapy Songwriting was not used in the wait-list group
11 How well: Planned fidelity strategies and assessment	Pre-designed fidelity checklist completed by music therapists every session Observer-rated fidelity checklist completed by independent music therapist rater
12 How well: Actual	Mean adherence of 44.45% (SD 25.94) with moderate reliability when coded by an independent rater. All manual components were used but with different sections occurring at different points in the therapy process

Adaptations to Grocke et al.’s intervention [33] included group members sharing pre-known songs in the early phases of the group; group improvisation after ice-breaker activities and before working on songs; and building time for the group to decide what they would like their end product to be (e.g. a compact disc (CD) or a group performance). Unlike Grocke et al. [33] who used a recording studio at the end of therapy, recording took place during the music therapy sessions using GarageBand software [48] and formed a major part of the group process.

Based on feedback from patient and carer groups, group music therapy took place in non-NHS premises in a community centre within one London borough. The centre offered facilities for additional social contact, such as a café and wider non-medical community groups. Sessions were provided three times per week over 14 weeks by two HCPC-registered music therapists. Sessions lasted 90 minutes and consisted of opening warm-up activities (such as passing an instrument), sharing current state (which, with permission, was written onto a flip chart for later lyric writing) and then moving into group improvisation. Music therapists transitioned into songwriting from this point, focusing on lyric creation, musical ideas or motifs and later recording. Opportunities were offered after each activity for verbal reflection. The last 15 minutes were dedicated to reviewing the session either through group discussion, or by playing music together.

### Wait-list control

The wait-list control group received treatment as usual for the study duration, which involved either psychopharmacological medication, psychological therapy or a combination. At the end of the final follow-up assessment, a further songwriting music therapy group was offered to these participants.

### Assessment measures

The purpose of a feasibility study is to determine whether or not it is possible to proceed with a given intervention or research design before moving to a larger scale [49]. In order to do this, it is recommended to establish pre-defined stop–go criteria [49] to aid the decision of whether or not to proceed. Whilst the criteria can vary from study to study, many take the format of a ‘traffic light’ system to aid identification of thresholds where a criterion is feasible (‘green’), not feasible (‘red’) or potentially feasible with modifications (‘amber’). Our pre-defined stop–go criteria were published in the study protocol [46] and are summarised in Table 2.

- Feasibility/acceptability of the research methodology (objectives 1–5)

**Table 2:** Stop–go feasibility criteria

Outcome	Method	Success criteria				Timing
		**Stop**	**Continue, modify protocol**	**Continue without modification but monitor closely**	**Continue without modifications**	
Acceptability of methodology	Recruitment & retention rates as below					End of recruitment (week 8)
	Compliance	Mean attendance < 10 sessions	Mean attendance < 14 sessions	Mean attendance 14 sessions	Mean attendance 14 + sessions	End of int. (week 22)
	End interviews	Unfavourable views, serious concerns	Unfavourable views, suggestions for modification	Favourable views, suggestions for modification	Favourable views, no concerns	1 month post-intervention (week 26)
Feasibility of recruitment processes	Screening rates	Identify < 50 potentially eligible subjects	Identify < 100 potentially eligible subjects	Identify 100–128 potentially eligible subjects	Identify > 128 potentially eligible subjects	End of recruitment
	Recruitment rates	Recruit < 50% of sample size	*N* < 25 in 8 weeks, < 5% per week	*N* = 25–30 in 8 weeks, < 13% per week	*N* = 30 in 8 weeks, 13% per week or greater	End of recruitment
	Participation rates	Participation rate < 5%	Participation rate 5–15%	Participation rate 15–25%	Participation rate 25% or greater	6 months post-intervention
	Retention rates	Attrition > 75%	Attrition 50–75%	Attrition 30–50%	Attrition < 30%	6 months post-intervention
	End interviews	N/A	Major suggestions to improve recruitment processes	Minor suggestions to improve recruitment processes	No suggestions to improve expressed	1 month post-intervention (week 26)
Identify N eligible participants, participant rates and retention rates	N identified by HCPs	< 50 identified	50–100 identified	100–128 identified	> 128 potentially eligible identified	End of recruitment
	N expressing interest	< 30 express interest	30–40 express interest	40–60 express interest	> 60 express interest	End of recruitment
	N providing consent	< 15 provide consent	15–25 provide consent	25–30 provide consent	30 provide consent	End of recruitment
	N lost to follow-up	Attrition > 75%	Attrition 50–75%	Attrition 30–50%	Attrition < 30%	1 month post-intervention, 3 and 6 months post-intervention
Researcher time and costs per participant	Researcher diary	N/A	Researcher time exceeds allocated time requiring additional study support	Researcher time and cost only just covers time required	Researcher time and cost fully covers time required	6 months post-intervention
Appropriate outcome measures	Variability of outcome Estimate of control mean and SD of change	No difference or clinically important difference favouring control detected based on confidence limits	Difference cannot be detected based on confidence limits but data suggests improvement favouring intervention	Difference can be detected based on confidence limits	Clinically important difference can be detected based on confidence limits	End of intervention
Intervention components	Therapist adherence	Adherence < 50%	Adherence < 50%	Adherence 50–75%	Adherence > 75%	End of intervention
	End interviews	Serious concerns expressed regarding intervention	Major suggestions to adapt intervention	Minor suggestions to adapt intervention	No concerns or suggestions to adapt intervention	
Intervention adherence	Therapist self-rated adherence Video rated adherence	Adherence < 25%	Adherence 25–50%	Adherence 50–75%	Adherence > 75%	End of intervention
Estimate of cost of intervention and services received	Therapist time CSRI	Cost significantly greater than usual care, no potential to modify intervention, no indication of benefits	Cost is greater than usual care—intervention may be modified, but outcomes suggest some benefits	Cost is greater than usual care but outcomes strongly suggest benefits	Cost is equivalent to or slightly greater than usual care, outcomes strongly suggest benefits	6 months post-intervention

Feasibility of recruitment processes (objective 1) and identification of the number of eligible participants, participation and retention rates (objective 2) were assessed through descriptive analysis of recruitment and drop-out rates and qualitative end interviews with participants and referring staff. Researcher time (objective 3) was assessed through researchers keeping logs of contact, dates of visits and time taken throughout the study. Outcome measure appropriateness (objective 4) was assessed by examining descriptive statistics and missing data. For clinical outcomes, our proposed primary endpoint was in the week following the intervention end (post-intervention), with secondary endpoints 3 and 6 months post-intervention. Acceptability of the research methodology to participants and patients (objective 5) was assessed through thematic analysis of qualitative interviews at the end of intervention.

(b) Feasibility/acceptability of the intervention (objective 6)

Feasibility/acceptability of the intervention (objective 6) was assessed through a nested process evaluation which aimed to understand (a) how the intervention was delivered in practice (treatment fidelity analysis), (b) describe processes of attendance and hypothesised process factors of self-reported depression, mood and group relationships from week to week and (c) understand subjective experiences and attributions for change of the intervention from the perspective of patients, music therapists and referring staff. To assess treatment fidelity, music therapist self-reported adherence to the manual each session and video analysis of 25% of sessions by independent raters (both music therapists) was collected using the same adherence proforma. To examine attendance and hypothesised process factors, group attendance, self-reported depression and weekly process measures of mood and group relationships were collected. For subjective experiences and change attributions, end of therapy interviews were conducted with patients and music therapists using the Client Change Interview [50]. This was adapted for referring staff and music therapists to reflect on changes observed in participants. Qualitative interviews were conducted by unblinded members of the research team and clinical studies officers supporting the study. Finally, as part of good clinical practice, adverse events were monitored throughout the study and were considered in relation to intervention safety and potential adverse outcomes.

(iii) Health service use (objective 7)

Health service use data were collected by examining medical records for any hospitalisation and using the Client Services Receipt Inventory at baseline, in the week following the intervention (post-intervention), 3 and 6 months post-intervention.

### Proposed primary symptom outcomes

Both observer-rated and self-report measures were used to assess depression symptoms.

#### Montgomery-Åsberg Depression Rating Scale (MADRS) [51]

The MADRS is an observer-rated 10-item scale known to be sensitive to change with good predictive validity for major depressive disorder [52]. Symptoms are rated from 0 (not present) to 6 (extreme problems) and summed to form a total score (0–60). Research Assistants were trained in its use with the accompanying interview guide (SIGMA [53]) prior to assessments with high inter-rater reliability (ICC = 0.995 (*p* < 0.001), 95% CI 0.987–0.999). Estimates for the minimal clinically important difference (MCID) range from a 1.6 to 1.9 change from baseline with remission cut-off at < 9 points [54, 55]. Bandelow et al. found scores ≤ 5 are symptom-free remission, ≤ 11 remission and a decrease in 39% from baseline corresponded to ‘much improved’ on the clinical global impressions scale [56, 57].

#### Beck Depression Inventory II (BDI-II) [58]

The BDI-II is a widely used self-reported 21-item measure of depression with good internal consistency, sensitivity to change and established cutoffs for minimal (raw score < 13), mild (14–19), moderate (20–28) and severe (29–63) depression [58]. Items are rated on a scale of 0 (no problems) to 3 (extreme problems), and summed to form a total score (0–63). The estimated MCID is estimated at either a reduction of 5 points [59, 60] or a 30% reduction in total score [61], 17.5% reduction in scores for depressed patients and 32% for those with a longer duration and non-response to antidepressants [62].

### Secondary and exploratory outcomes

#### Brief Symptom Inventory (BSI) [63]

The BSI is a widely used 53-item self-report measure of psychological distress with good internal consistency and established outpatient norms in both United States and UK samples [63, 64]. Symptoms are rated on a Likert scale from 0 (not at all) to 4 (extremely). There are nine subscales for symptom clusters (0–4) and three global indices of distress; global severity index, positive symptom distress index and positive symptom total, of which global severity is used as a single summary measure.

#### Rosenberg self-esteem scale (RSES) [65]

The RSES is a widely used 10-item self-report measure of self-esteem. Items are rated on a 4-point Likert scale from ‘strongly agree’ to ‘strongly disagree’. Four items are reverse scored, and item totals are summed (0–40). The scale has good internal consistency (0.68–0.86) [66] and construct validity [67].

#### General Perceived Self-efficacy Scale (GPSES) [68]

The GPSES is a 10-item self-report measure of personal agency, rated on a 4-point Likert scale from ‘not at all true’ to ‘exactly true’. Item totals are summed (10–40). The scale has confirmed uni-dimensionality and good internal consistency (0.82–0.93) [68].

#### Client satisfaction questionnaire (CSQ) [69]

The CSQ measures self-reported satisfaction with services and is rated on an 8-item scale from 1 (dissatisfied) to 4 (very satisfied) and items summed (8–32). The scale is widely used in health services research and has good internal consistency (0.83–0.93) [69].

#### Work and social adjustment scale (WSAS) [70]

The WSAS is a self-report 5-item scale that measures the degree to which work and social life are impaired due to a health condition. Items are rated on an 8-point scale from 0 (not at all impaired) to 8 (very severely impaired). Item totals are summed (0–40). The scale has demonstrated internal consistency (0.70–0.94) and a test–retest correlation of 0.73 [70].

#### Manchester Short Quality of Life scale (MANSA) [71]

The MANSA is a 16-item self-report scale measuring satisfaction with different areas of life. Twelve items are rated on a 7-point Likert scale ranging from 1 (‘couldn’t be worse’) to 7 (‘couldn’t be better) which are summed (12–84). Four items are dichotomous (yes/no) to indicate whether the person has a close friend, saw a friend in the last week, was accused of a crime or was a victim of physical violence. The scale has good internal consistency (0.74) and correlations of 0.83 and higher with the longer Lancashire Quality of Life Profile [71].

#### Life Skills Profile (LSP) [72]

The LSP is an observer-rated 39-item profile, originally designed for patients with schizophrenia. Various domains of social functioning are rated on a 4-point scale from no difficulty (4) to considerable difficulty (1). Items are summed into five subscales: self-care, non-turbulence, social contact, communication and responsibility and overall functioning score (39–154). Internal consistency ranges from 0.67 to 0.88, and the scale demonstrated good sensitivity to change in community patients with chronic mental illness within an assertive outreach service [73].

#### Level of hospitalisation

Psychiatric hospital admissions, length of stay and readmissions were recorded from medical records for the purposes of this study.

#### Client services receipt inventory (CSRI) [74]

The CSRI was used to collect information on face-to-face professional contacts, use of day care services, contact with police, medications, time off work/college and receipt of state benefits.

### Process measures

Within the treatment arm, process measures of mood and group relationships were administered once per week pre- and post-session. In addition, the BDI-II was completed post-session in weeks 3, 6, 9 and 12 of the intervention to track any self-reported changes in depression during the intervention period. Attendance was logged by the therapist at every session, and reasons for non-attendance were recorded. Finally, qualitative end of therapy interviews were completed with participants in both treatment and wait-list groups. These interviews were optional for participants.

#### Dispositional Mood Scale (DMS) [75, 76]

The DMS is a self-report scale consisting of 20 adjectives describing current internal states. Adjectives are rated on a scale of 1 (very slightly or not at all) to 5 (extremely) and summed as four subscales of positive energy, tiredness, negative activation and relaxation. A further two-factor solution is possible: ‘Pleasant-Activation, Unpleasant Deactivation’ and ‘Unpleasant activation, Pleasant deactivation’. Internal consistency varies between α: 0.83 and 0.93 [75].

#### Relationship Satisfaction Scale (RSS) [77]

The RSS is a 7-item self-report scale assessing the quality of a relationship. Items are rated on a 7-point Likert scale from 0 (‘very dissatisfied’) to 6 (‘very satisfied’) and summed to form an overall satisfaction score. The scale has not been validated, but assessed domains of relevance to group relationships (e.g. communication and openness, conflict resolution, intimacy and closeness).

#### Music therapy group attendance

Attendance was recorded by the music therapists every session on a pre-designed proforma, including space to record reasons for non-attendance.

#### Experience of therapy and research incorporating adapted client change interview [50]

A topic guide was pre-designed to enquire about experiences of both the therapy and taking part in the study in qualitative interviews. For participants in the treatment arm, the Client Change Interview [50] was used to explore helpful and hindering factors in therapy, changes experienced during therapy and attributions for this.

#### Adverse events

Adverse events were recorded from the point of written informed consent to 7 days post-cessation of the study. Active monitoring commenced from the first point of attendance of group music therapy to 1 week after the intervention finished. Expected adverse events were defined as:

- A participant exhibiting aggression (nonverbal or verbal behaviour)
- A participant causing harm to another person
- Disclosure of thoughts or plans which may place the individual or others at risk of harm.

Serious adverse events that were defined for this study context included:

- A participant making a suicide attempt
- A participant causing life threatening injury to another
- An event occurring during the course of the study which resulted in hospitalisation or prolongation of existing hospitalisation related to their mental health.

### Rationale for sample size

Papers considering sample size for feasibility studies suggest inclusion of upwards of 24–50 participants [78–80]. As the feasibility of our recruitment processes and sample were unknown, we based our sample size around what was practicable to provide within the study timeframe. We aimed to recruit 30 patients to participate in three groups of 10 patients in each. Participation rates in similar studies were between 25% and 33% of eligible patients consenting [81–83]. A sample size of 30 would allow us to estimate a participation rate of 25% to within 95% confidence interval of + / − 15%. We estimated 1300 patients would be eligible within primary care (assuming one fifth of those with current depression) and that each practice in the locality would therefore have around 20 with enduring symptoms. Secondary care services reported around 1960 patients with a diagnosis of depression, suggesting 392 would be potentially eligible for this study. Assuming a participation rate of 25%, we aimed to approach 128 patients, with the aim of recruiting 4 per week over 8–10 weeks.

### Randomisation

To gain sufficient information regarding the intervention, we used an imbalanced design, randomising 20 participants to group music therapy and 10 to the wait-list control. We used simple block randomisation once all 30 participants were recruited and baseline measures completed. Randomisation was generated by a researcher independent to the study team, using the Experimental Design Generator and Randomiser (EDGAR-II) [84]. One unblinded study team member and music therapists were informed of the allocation, who then informed participants.

### Blinding

Researchers conducting assessments and the co-Chief Investigator (Priebe) were blinded to participant allocation. Due to the trial design, participants, music therapists and the clinical teams were not blinded to allocation. One Chief Investigator (Carr) and Clinical Studies Officers were unblinded to enable communication with clinicians and administration of process measures.

To maintain blinding of researchers, it was explained to participants on allocation that it was important not to reveal this to the researcher who had conducted their assessments. Participants were reminded in every communication from researchers not to mention whether they had received music therapy or not.

### Analysis

For research methodology feasibility measures (objectives 1–4), we calculated screening, recruitment and drop-out rates, distributions of baseline characteristics and all outcomes 1 week and 3 and 6 months post-intervention. Clinical outcomes were analysed as intention-to-treat, using mean scores for each group and 95% confidence intervals. We then used a mixed linear model, adjusting for baseline scores of the given outcome and any significant baseline characteristics. The intra-cluster correlation coefficient was calculated for group clustering. Adverse events were categorised and reported for each trial arm.

For intervention feasibility measures (objective 6), we explored using descriptive statistics, any differences between compliant/non-compliant attenders, responders and non-responders. Qualitative interviews were analysed in two stages. In the first stage, participants who had received music therapy were analysed to explore their experiences of the intervention and any changes (objective 6) using interpretative phenomenological analysis [85]. This enabled us to gain an in-depth understanding of participants’ experiences during the songwriting groups including the meaning attributed by participants to their experiences. Further details of the analysis and findings are published in full elsewhere [85]. In a second stage, given the larger number of interviews and pre-defined format of research procedures, comments relating to acceptability and experiences of research procedures (objective 1) were analysed using deductive coding against each element of the research design and then grouped to form a basic thematic analysis [86]. For health service use (objective 7), hospitalisation and use of services were examined descriptively and compared between groups.

## Results

### Feasibility and acceptability of research methodology (objectives 1–5)

#### Recruitment

**Fig. 1:**
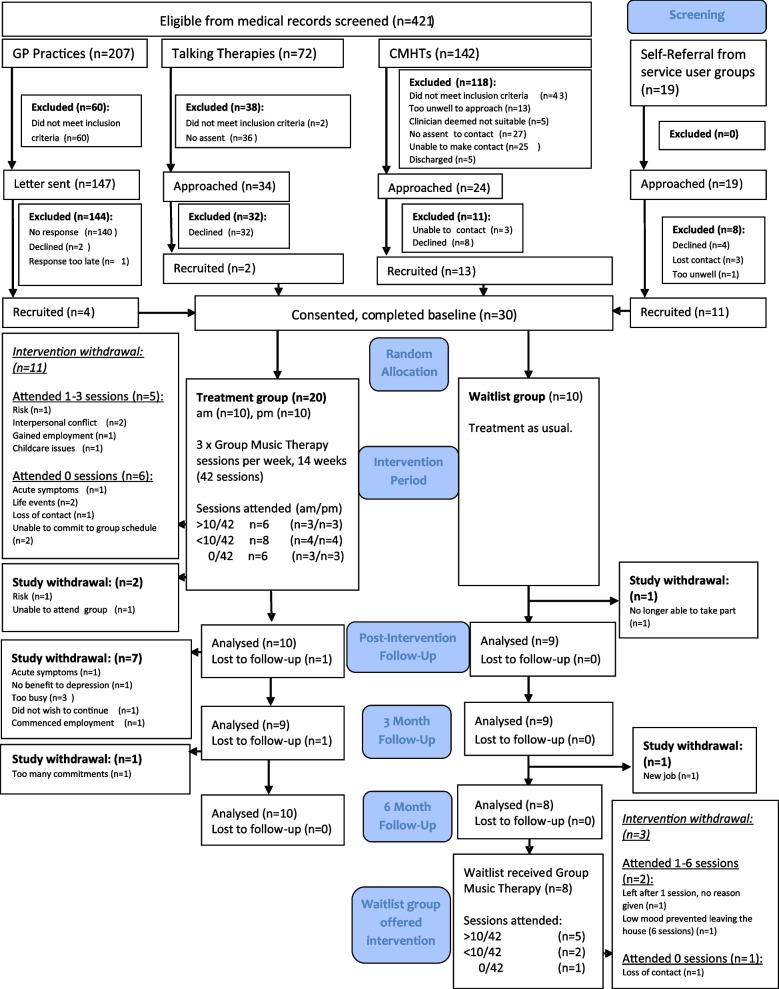
CONSORT diagram

Flow of participants in the study is shown in the Consolidated Standards of Reporting Trials (CONSORT) diagram (Fig. 1) and baseline characteristics in Table 3. A total of 421 patients were screened and 235 potentially eligible participants identified. Reasons for exclusion at this stage were not meeting the inclusion criteria (*N* = 105), no clinician assent for contact (*N* = 63), researchers unable to make contact (*N* = 25) or participants being deemed too unwell to approach (*N* = 13) or unsuitable by clinicians (*N* = 5). Five were discharged from services before they could be approached. Of the 235 participants approached, 83 expressed interest with a participation rate (from potentially eligible participants) of 12.7%. Forty-six declined whilst 146 were unable to contact or did not respond. One GP practice participant expressed interest but was too late to join the study, and one self-referred participant was too unwell to recruit within the study window. Whilst there were equivalent numbers of potentially eligible participants within GP and Community Mental Health settings, recruitment was most successful via Community Mental Health teams (CMHT) and self-referral from public engagement events. The recruitment target was achieved, with 30 participants providing informed consent over an 8-week period and recruitment rate of 12.5% (Table 4). Recruitment was initially slow with six participants recruited in the first 4 weeks and recruitment then peaking in weeks 5 (9 recruited) and 8 (5 recruited) (Table 4). Researcher time was adequate to cover the necessary research tasks over the course of the study.

**Table 3:** Baseline socio-demographic and clinical characteristics

**Baseline characteristics**	Treatment group (*n* = 20)	Wait-list group (*n* = 10)	Total (*n* = 30)
Age	42.25 (37.09, 47.41)	48.8 (42.07, 55.53)	44.43 (40.39, 48.47)
Females:Males^a^	13:7	3:7	16:14
English First language:Second language	10:10	8:2	18:12
In Employment:Unemployed	4:16	1:9	5:25
Primary diagnosis			
F31	5/20	1/10	6/30
F32	3/20	2/10	5/30
F33	7/20	5/10	12/30
F41	3/20	0/10	3/30
F43	2/20	2/10	4/30
Duration diagnosis (years)	9.80 (4.37, 15.23)	12.5 (4.14, 20.86)	10.70 (6.41, 15.00)
Hospitalised in the last year	6/20	1/10	7/30
Medication			
Antidepressants	13/20	6/10	19/30
SNRI	4/20	1/10	5/30
TCA	6/20	0	6/30
NASSA	5/20	0	5/30
SSRI	4/20	5/10	9/30
Antipsychotic	14/20	3/10	17/30
Atypical	13/20	3/10	16/30
Typical	1/20	0	1/30
Hypnotics/Anxiolytics	7/20	2/10	9/30
Benzodiazapine	1/20	0	1/30
Antihistamine	5/20	1/10	6/30
Hypnotic	1/20	1/10	2/30
Mood stabilisers	2/20	1/10	3/30
No psychiatric medication	3/20	3/10	6/30
Previous receipt of music therapy	1/20	2/10	3/30
Interest in Music − ve	3.3 (2.74, 3.87)	2.85 (2.24, 3.46)	3.15 (2.74, 3.56)
Interest in Music + ve	3.35 (3.03, 3.68)	3.65 (3.13, 4.17)	3.45 (3.19, 3.71)
MADRS	25.85 (21.61, 30.09)	19.2 (10.73, 27.67)	23.63 (19.76, 27.50)
BDI-II	30.92 (25.69, 36.15)	23.56 (13.35, 33.77)	28.47 (23.78, 33.15)
CSQ	24.15 (21.57, 26.73)	22.20 (17.92, 26.48)	23.5 (21.39, 25.61)
MANSA	3.64 (3.20, 4.07)	4.03 (3.44, 4.61)	3.77 (3.43, 4.10)
RSES	22.3 (20.21, 24.59)	24.2 (20.67, 27.73)	22.93 (21.20, 24.67)
GPSES^b^	22.05 (18.97, 25.13)	26.4 (23.27, 29.53)	23.5 (21.18, 25.82)
WASAS	26.85 (23.03, 30.67)	21.80 (13.22, 30.38)	25.17 (21.54, 28.79)
BSI Somatisation^e^	2.44 (2.06, 2.81)	1.15 (0.53, 1.78)	2.00 (1.63, 2.39)
BSI Obsessive–Compulsive	2.65 (2.31, 2.99)	2.19 (1.54, 2.84)	2.50 (2.19, 2.80)
BSI Interpersonal Sensitivity^f^	2.46 (2.07, 2.85)	1.28 (0.55, 2.00)	2.06 (1.68, 2.45)
BSI Depression^g^	2.67 (2.27, 3.07)	1.81 (1.03, 2.58)	2.38 (2.00, 2.76)
BSI Anxiety^h^	2.31 (1.95, 2.68)	1.44 (0.80, 2.07)	2.02 (1.68, 2.36)
BSI Hostility^i^	2.56 (2.06, 3.05)	1.68 (1.25, 2.12)	2.27 (1.89, 2.64)
BSI Phobia^j^	2.51 (2.18, 2.83)	1.42 (0.47, 2.36)	2.14 (1.75, 2.54)
BSI Paranoia^k^	2.52 (1.99, 3.06)	1.77 (1.21, 2.33)	2.27 (1.86, 2.67)
BSI Psychoticism	2.72 (2.26, 3.19)	2.01 (1.25, 2.77)	2.49 (2.09, 2.88)
BSI Global Severity Index^l^	2.04 (1.65, 2.44)	1.12 (0.59, 1.66)	1.74 (1.39, 2.08)
BSI Positive Symptom Total^m^	39.90 (34.86, 44.94)	29.20 (20.27, 38.13)	36.33 (31.75, 40.91)
BSI Positive Symptom Distress Index^n^	2.58 (2.27, 2.91)	1.85 (1.39, 2.30)	2.34 (2.06, 2.62)
LSP Self care^c^	32.2 (30.48, 33.92)	35.2 (32.89, 37.51)	33.2 (31.80, 34.61)
LSP Non-turbulence	40.30 (36.75, 43.85)	42.70 (40.59, 44.81)	41.1 (38.69, 43.51)
LSP Social contact	14.90 (13.27, 16.54)	15.60 (13.01, 18.20)	15.13 (13.83, 16.44)
LSP Communication^d^	20.05 (18.71, 21.39)	22.10 (21.12, 23.08)	20.73 (19.75, 21.71)
LSP Responsibility	17.35 (16.04, 18.66)	18.30 (16.72, 19.88)	17.67 (16.69, 18.64)

^a^Wilcoxon-Mann–Whitney, *z* = –2.096, *p* = .04

^b^Two-tailed *t*-test, unequal variances assumed, *p* = .04

^c^Two-tailed *t*-test, unequal variances assumed, *p* = .03

^d–n^Two-tailed *t*-test, unequal variances assumed

^d^*p* = .01

^e^*p* < .01

^f^*p* < .01

^g^*p* = .05

^h^*p* = .02

^i^*p* < .01

^j^*p* = .03

^k^*p* = .05

^l^*p* < .01

^*m*^*p* = .03

^n^*p* < .01

Baseline data: mean (95% confidence interval)

**Table 4:** Weekly recruitment rates

Week	*N* recruited	Cumulative total	Weekly percentage	Cumulative percentage
1	0	0	0%	0%
2	1	1	3%	3%
3	3	4	10%	13%
4	2	6	7%	20%
5	9	15	30%	50%
6	6	21	20%	70%
7	3	24	10%	80%
8	6	30	20%	100%

#### Baseline characteristics

Participants were on average 44 years old, with the majority holding a diagnosis of recurrent depressive disorder (ICD10 F33.0, 12/30 participants). Mean duration of diagnosis was 10.7 years (range, 1–40 years). Few (3/30) had previously attended music therapy. Groups differed significantly at baseline regarding gender (65% of the treatment arm were female compared to 30% in the wait-list arm), self-efficacy, BSI scores and life skills of self-care and communication. The treatment arm also had a greater proportion of participants with English as a second language. Depression symptom severity had high variance, with participants scoring a large range of the MADRS (0–48), and BDI-II (1–48). Two wait-list participants met the criteria for remission at baseline (< 9) on the MADRS, whilst seven met criteria for mild or moderate depression on the BDI-II (3 in treatment, 4 in wait-list).

#### Retention

Ten participants withdrew from the study between allocation and post-intervention with 60% retention (*n* = 18) at 6-month follow-up. On allocation, one wait-list participant withdrew due to no longer being able to take part. The remaining nine withdrawals were in the treatment arm, of which six did not attend any sessions. Those who did not attend withdrew from both study and intervention due to being unable to commit to the group schedule (*n* = 2), life events (*n* = 2), symptom severity (*n* = 1) and loss of contact (*n* = 1). Of those who did attend, one was withdrawn due to risk after the first session, one felt that the study was not of benefit to depression after four sessions and one felt further study participation was invalid having only attended three sessions and gained employment. At 3 months follow-up, one further treatment participant who did not attend any sessions withdrew due to too many other commitments and one wait-list participant due to commencing employment. Outside of withdrawals, two separate losses to follow-up occurred, once at 3 months and once at 6 months in the treatment arm.

#### Blinding

There were four instances of unblinding. One post-allocation, where an intervention participant called the researcher to inform of the outcome; twice when arranging 1 week post-intervention assessments with intervention participants and one wait-list participant at the 6-month follow-up. In the three cases of scheduling assessments, all were due to participants sharing upcoming intervention-based appointments. With two blinded team members, there was capacity within the research team to cover these assessments enabling all assessments to be completed with blinding intact.

#### Clinical outcomes

Raw and adjusted outcomes are shown in Tables 5 and 6 respectively.

**Table 5:** Raw outcomes post-intervention and 3 and 6 months post-intervention

	Post-intervention raw scores						3-month raw scores						6-month raw scores					
	Treatment group *N* = 10			Wait-list group *N* = 9			Treatment group *N* = 9			Wait-list group *N* = 9			Treatment group *N* = 10			Wait-list group *N* = 8		
	Mean	SD	95%CI	Mean	SD	95%CI	Mean	SD	95%CI	Mean	SD	95%CI	Mean	SD	95%CI	Mean	SD	95%CI
MADRS	33.60	8.91	27.23, 39.97	23.44	13.89	12.76, 34.12	21.67	9.12	14.65, 28.68	21.44	12.08	12.16, 30.73	25.70	8.98	19.27, 32.13	22.00	10.81	12.96, 31.04
BDI-II	39.18	8.32	33.23, 45.13	25.29	11.43	16.51, 34.07	33.78	15.08	22.18, 45.37	26.28	11.89	17.14, 35.41	35.70	13.81	25.82, 45.58	26.89	14.50	14.77, 39.01
CSQ	21.80	6.11	17.43, 26.17	20.78	6.65	15.67, 25.89	22.22	8.06	16.03, 28.42	22.22	6.74	17.04, 27.40	23.60	8.68	17.39, 29.81	18.88	5.59	14.20, 23.55
MANSA	2.90	0.85	2.29, 3.51	3.95	0.97	3.21, 4.70	3.43	1.22	2.49, 4.36	4.07	1.03	3.28, 4.86	3.24	0.85	2.63, 3.85	3.86	1.47	2.63, 5.08
RSES	18.20	4.98	14.63, 21.77	23.78	4.09	20.64, 26.92	22.22	7.61	16.37, 28.07	24.67	4.72	21.04, 28.29	21.10	7.61	15.66, 26.54	25.13	5.57	20.47, 29.78
GPSES	21.50	8.13	15.69, 27.31	26.11	4.31	22.80, 29.43	24.56	7.97	18.43, 30.68	26.22	4.79	22.54, 29.90	22.30	7.20	17.15, 27.45	24.13	5.38	19.62, 28.63
WSAS	31.10	6.08	26.75, 35.45	21.56	10.56	13.44, 29.67	30.22	11.51	21.38, 39.07	23.67	9.21	16.59, 30.74	30.60	4.72	27.22, 33.98	22.50	10.31	13.88, 31.12
BSI SOM	2.67	0.88	2.04, 3.30	1.50	0.74	0.94, 2.07	1.86	1.08	1.03, 2.69	1.46	0.70	0.92, 2.00	2.10	1.00	1.39, 2.82	1.32	0.69	0.75, 1.90
BSI OC	3.02	0.82	2.44, 3.61	2.13	0.72	1.57, 2.68	2.59	1.03	1.80, 3.39	2.01	0.84	1.37, 2.66	2.78	0.78	2.22, 3.34	2.21	0.91	1.45, 2.97
BSI IP	2.93	0.85	2.32, 3.53	1.62	1.00	0.85, 2.39	2.68	1.13	1.81, 3.54	2.25	0.81	1.63, 2.87	2.58	0.99	1.87, 3.28	1.80	0.96	1.00, 2.60
BSI DEP	2.98	0.94	2.31, 3.65	1.88	1.11	1.02, 2.73	2.84	1.17	1.94, 3.74	2.16	0.93	1.45, 2.88	2.74	1.15	1.91, 3.56	2.19	0.97	1.38, 3.00
BSI ANX	2.40	0.71	1.89, 2.91	1.57	0.97	0.83, 2.32	2.16	1.20	1.23, 3.08	1.87	0.79	1.27, 2.48	2.36	0.81	1.78, 2.94	1.71	0.71	1.12, 2.31
BSI HOS	2.12	0.93	1.45, 2.79	1.51	1.16	0.62, 2.40	2.01	1.09	1.16, 2.85	1.70	0.79	1.09, 2.31	2.25	1.04	1.50, 2.99	1.49	0.93	0.71, 2.27
BSI PHO	2.76	0.64	2.31, 3.22	1.49	1.16	0.60, 2.38	2.48	1.09	1.64, 3.32	1.55	1.08	0.72, 2.39	2.52	1.05	1.76, 3.27	1.74	0.97	0.93, 2.55
BSI PAR	2.81	0.85	2.20, 3.42	1.69	0.62	1.22, 2.17	2.78	0.90	2.09, 3.47	1.90	0.72	1.34, 2.46	2.68	0.87	2.06, 3.30	1.75	0.82	1.06, 2.44
BSI PSY	3.03	0.87	2.41, 3.65	1.89	1.25	0.93, 2.85	2.99	1.27	2.02, 3.96	1.80	1.15	0.91, 2.68	2.91	0.90	2.26, 3.55	2.36	1.09	1.45, 3.28
BSI GSI	2.41	0.79	1.85, 2.97	1.25	0.73	0.68, 1.81	2.15	0.91	1.45, 2.85	1.29	0.73	0.73, 1.85	2.28	0.87	1.66, 2.90	1.31	0.68	0.74, 1.88
BSI PST	44.40	7.46	39.07, 49.73	31.89	13.01	21.89, 41.89	40.44	15.23	28.74, 52.15	32.78	10.99	24.33, 41.22	44.20	12.79	35.05, 53.35	33.38	11.39	23.85, 42.90
BSI PSDI	2.80	0.73	2.28, 3.32	1.92	0.51	1.52, 2.31	2.61	0.71	2.06, 3.16	1.94	0.64	1.44, 2.43	2.62	0.67	2.14, 3.09	1.95	0.70	1.36, 2.54
LSP CAR	30.30	2.95	28.19, 32.41	34.44	3.50	31.75, 37.14	34.44	2.83	32.27, 36.62	34.33	3.43	31.70, 36.97	35.30	2.11	33.79, 36.81	33.75	3.69	30.66, 36.84
LSP NON	38.80	5.33	34.99, 42.61	43.00	2.18	41.32, 44.68	43.67	3.81	40.74, 46.59	44.89	2.62	42.88, 46.90	45.40	2.27	43.78, 47.02	45.13	3.27	42.39, 47.86
LSP SOC	13.50	4.93	9.98, 17.02	15.44	4.48	12.00, 18.88	14.56	4.82	10.85, 18.26	14.56	5.17	10.58, 18.53	15.90	4.12	12.95, 18.85	15.13	4.42	11.43, 18.82
LSP COM	21.40	2.01	19.96, 22.84	22.78	1.20	21.85, 23.70	22.22	1.92	20.74, 23.70	22.33	1.22	21.39, 23.27	22.20	0.92	21.54, 22.86	22.38	1.60	21.04, 23.71
LSP RES	16.40	1.58	15.27, 17.53	18.44	1.01	17.67, 19.22	18.44	1.59	17.22, 19.67	17.67	1.41	16.58, 18.75	18.20	2.04	16.74, 19.66	18.50	1.20	17.50, 19.50
LSP SUM	120.4	8.28	114.5, 126.3	134.11	10.14	126.3, 141.9	133.3	9.72	125.9, 140.8	133.8	9.88	126.2, 141.4	137.0	6.43	132.4, 141.6	134.9	9.20	127.2, 142.6

**Table 6:** Outcomes post-intervention and 3 and 6 months post-intervention adjusted for baseline characteristics

	Post-intervention						3 months						6 months					
	Treatment group *N* = 10			Wait-list group *N* = 9			Treatment group *N* = 9			Wait-list group *N* = 9			Treatment group *N* = 10			Wait-list group N = 8		
	Mean	95% CI		Mean	95% CI		Mean	95% CI		Mean	95% CI		Mean	95% CI		Mean	95% CI	
MADRS	31.28	25.03	37.53	25.51	18.95	32.08	19.82	13.36	26.28	23.51	17.04	29.98	24.91	18.79	31.03	23.31	16.46	30.16
BDI-II	35.87	30.03	41.71	28.61	22.46	34.75	30.72	22.97	38.48	29.60	21.83	37.36	34.08	27.30	40.85	29.03	21.45	36.62
CSQ	21.36	17.48	25.24	21.41	17.31	25.51	21.46	16.84	26.08	22.86	18.24	27.47	22.56	17.69	27.43	20.17	14.71	25.62
MANSA	3.35	2.87	3.83	3.43	2.92	3.94	3.89	3.59	4.20	3.55	3.24	3.85	3.67	3.19	4.16	3.41	2.87	3.96
RSES	19.45	17.53	21.37	22.31	20.28	24.34	23.73	21.17	26.28	23.20	20.65	25.75	21.95	19.45	24.46	24.10	21.30	26.90
GPSES	22.93	20.14	25.72	25.01	22.07	27.95	25.20	22.44	27.96	25.12	22.35	27.88	22.94	20.52	25.37	23.29	20.58	26.01
WSAS	27.82	24.20	31.44	24.96	21.14	28.77	27.71	21.59	33.83	27.07	20.92	33.22	28.69	24.94	32.45	24.16	19.98	28.34
BSI SOM	2.08	1.65	2.51	2.09	1.64	2.54	1.36	0.81	1.90	2.04	1.49	2.60	1.73	1.29	2.17	1.78	1.29	2.28
BSI OC	2.84	2.36	3.32	2.28	1.78	2.78	2.47	1.91	3.04	2.17	1.60	2.74	2.70	2.20	3.21	2.33	1.76	2.89
BSI IIS	2.28	1.87	2.68	2.26	1.84	2.68	2.15	1.44	2.85	2.89	2.18	3.59	2.18	1.89	2.46	2.28	1.96	2.60
BSI DEP	2.50	2.03	2.96	2.37	1.88	2.87	2.40	1.84	2.97	2.66	2.09	3.23	2.42	1.97	2.86	2.57	2.07	3.06
BSI ANX	2.07	1.66	2.47	1.84	1.41	2.26	1.96	1.35	2.56	2.14	1.53	2.75	2.24	1.89	2.59	1.92	1.53	2.31
BSI HOS	1.74	1.24	2.24	1.88	1.35	2.40	1.71	1.29	2.14	2.07	1.64	2.50	2.00	1.44	2.56	1.77	1.15	2.40
BSI PHOB	2.34	2.01	2.67	1.89	1.55	2.24	2.14	1.67	2.60	1.96	1.49	2.43	2.25	1.84	2.66	2.09	1.63	2.55
BSI PAR	2.31	1.90	2.73	2.16	1.73	2.60	2.36	2.04	2.68	2.37	2.05	2.69	2.39	2.11	2.67	2.15	1.83	2.47
BSI PSY	2.57	2.13	3.01	2.39	1.92	2.85	2.56	2.02	3.11	2.29	1.75	2.84	2.56	2.18	2.94	2.74	2.32	3.16
BSI GSI	1.87	1.57	2.17	1.77	1.45	2.08	1.71	1.36	2.05	1.82	1.47	2.16	1.95	1.69	2.21	1.73	1.44	2.02
BSI PST	38.85	34.16	43.53	38.21	33.25	43.17	35.37	30.12	40.62	39.10	33.80	44.40	39.73	35.06	44.39	37.40	32.21	42.59
BSI PSDI	2.37	2.12	2.62	2.31	2.05	2.57	2.27	2.00	2.54	2.33	2.06	2.60	2.39	2.17	2.60	2.27	2.03	2.51
LSP CAR	31.11	29.15	33.06	33.67	31.62	35.73	35.12	33.68	36.57	33.56	32.11	35.01	35.82	34.05	37.60	33.06	31.08	35.05
LSP NON	39.18	36.86	41.50	42.75	40.31	45.19	43.89	42.12	45.66	44.64	42.87	46.42	45.46	43.26	47.66	44.89	42.43	47.35
LSP SOC	13.45	11.56	15.33	15.67	13.68	17.66	13.93	11.37	16.49	14.78	12.22	17.34	15.47	13.34	17.59	15.93	13.56	18.31
LSP COM	21.69	20.53	22.85	22.59	21.37	23.81	22.31	21.41	23.22	22.14	21.24	23.05	22.30	21.55	23.04	22.22	21.39	23.06
LSP RESP	16.52	15.69	17.34	18.35	17.49	19.22	18.50	17.56	19.45	17.57	16.63	18.52	18.26	17.01	19.52	18.42	17.02	19.83
LSP SUM	122.34	117.45	127.23	132.68	127.55	137.81	134.23	129.26	139.20	132.34	127.36	137.33	137.60	132.12	143.08	133.91	127.78	140.05

#### Primary outcome—MADRS

Groups differed at baseline (treatment 25.85, wait-list 19.20) with greater severity in the treatment group. Measures indicated a worsening of symptoms in both groups post-intervention (treatment 31.28; wait-list 25.51), with the treatment group then improving to better than baseline at 3- and 6-month follow-ups (3 month 19.82; 6 month 24.91). The wait-list group scored higher than baseline scores at 3 and 6 months (3 months: 23.51; 6 month 23.31). The intra-class correlation coefficient, demonstrating the level of clustering between groups was 0.088.

**Fig. 2:**
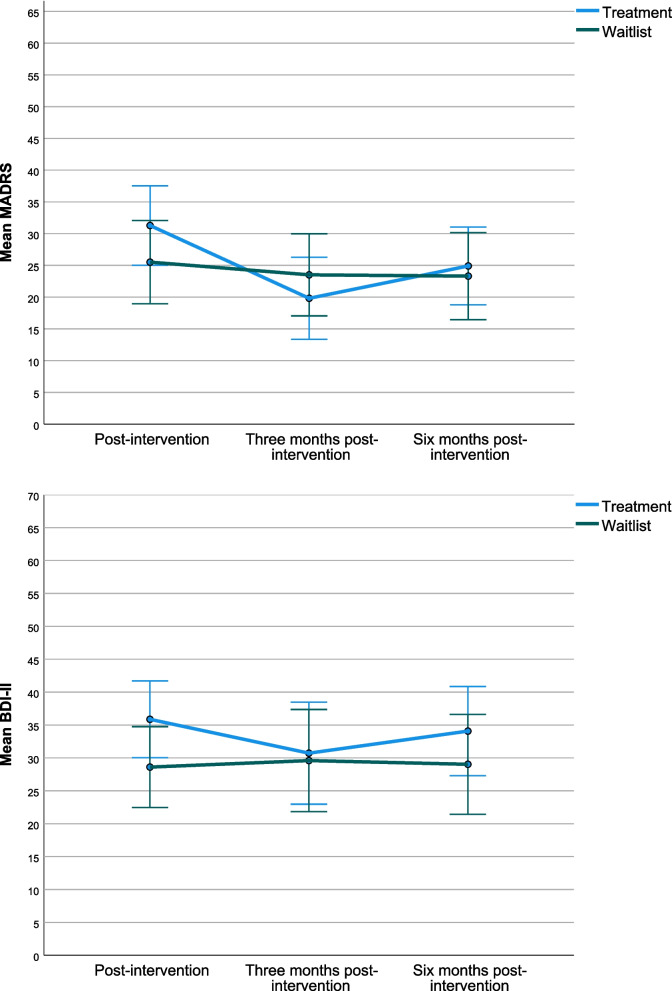
Estimated marginal means of MADRS and BDI-II outcome measures adjusting for baseline score

After adjusting for baseline scores, a change of greater than the MCID (− 5.04, reduction of 20.2% from baseline score) was seen at 3 months in the treatment group but not at 1 week or 6 months post-intervention (Fig. 2). Four participants in each arm saw reductions of more than 39%, equating to ‘much improved’ on the Clinical Global Impressions scale. For the four treatment participants, this was 3 and 6 months post-intervention. For the four wait-list participants, this was across all follow-up timepoints. Three participants qualified for remission (scores less than 9): One participant in the treatment arm (compliant attender) qualified as complete remission (< 5) and two in the wait-list arm (< 9). Both the wait-list participants in remission withdrew from the study at the point of offer of music therapy.

#### Secondary outcomes

Treatment group scores were worse compared to the wait-list group on all secondary measures 1 week post-intervention apart from BSI Somatisation (treatment: 2.08; wait-list 2.09) and BSI Hostility (treatment 1.74; wait-list 1.88). In the treatment group, mean difference improvements from baseline to 1 week post-intervention were seen in self-efficacy (+ 0.88), LSP communication (+ 1.64) and BSI subscales of somatisation (-0.36), interpersonal sensitivity (− 0.18), depression (− 0.17), anxiety (− 0.24), hostility (− 0.82), phobia (− 0.17), paranoia (− 0.21), psychosis (− 0.15), global severity (− 0.17), positive symptom totals (− 1.05) and positive symptom distress (− 0.22). In the wait-list group, all scales scored worse in mean differences from baseline to 1 week post-intervention apart from LSP subscales of non-turbulence (+ 0.05), social communication (+ 0.07), communication (+ 0.49) and responsibility (+ 0.05).

At 3 months, treatment group scores were more favourable compared to the wait-list group on all measures except the BDI-II (treatment 30.72; wait-list 29.60), CSQ (treatment 21.46; wait-list, 22.86) and WSAS (treatment 27.71, wait-list, 27.07). The treatment group showed mean difference improvements compared to baseline on all measures apart from CSQ (− 2.69), WSAS (+ 0.86) and LSP social contact (− 0.97). The wait-list group showed mean difference deterioration compared to baseline on all measures apart from satisfaction (+ 0.66), LSP non-turbulence (+ 1.94), LSP communication (+ 0.04) and BSI Obsessive–Compulsive subscale (− 0.02).

At 6 months, scores favoured the treatment group on CSQ (treatment, 22.56; wait-list, 20.17), MANSA (treatment, 3.67; wait-list 3.41), BSI subscales of somatisation (treatment 1.73; wait-list 1.78), interpersonal sensitivity (treatment 2.18, wait-list, 2.28), depression (treatment 2.42; wait-list 2.57), psychoticisim (treatment 2.56; wait-list, 2.74) and LSP self-care (treatment 35.82; wait-list 33.06), non-turbulence (treatment 45.46; wait-list 44.89), Communication (treatment 22.30, wait-list, 22.22) and LSP sum score (treatment 137.60; wait-list 133.91). Mean difference change compared to baseline was favourable on all measures apart from BDI-II (+ 3.08), Satisfaction (− 1.59), Self-esteem (− 2.25), WSAS (+ 6.89) and BSI Obsessive–Compulsive (+ 0.51). Wait-list mean difference scores deterioriated compared to baseline on all measures apart from the LSP sum score and subscales (LSP SUM + 0.01).

A negative MCID was detected 1 week post-intervention for the treatment arm after adjusting for baseline scores in the BDI-II (gain of 5.26). A positive BDI-II MCID was detected in three treatment group and four wait-list group participants via reduction of 5 + points, whilst two treatment and four wait-list participants had reductions of > 30%. Two treatment participants and five wait-list participants met criteria for ‘minimal’ depression.

### Acceptability of research methodology to professionals and patients (objective 5)

End interviews with 10 participants and 7 clinical staff indicated generally good acceptability of the research methodology and study procedures. Clinicians stated that the referral process had been easy. Referrers were positive about the intervention being offered, particularly its intensity and opportunities for socialisation and enjoyment. One suggested that it had been a reminder that more was available than cognitive behavioural therapy (CBT). Patients declined participation mostly due to not being interested or to the time commitment of attending groups. Clinicians valued researchers being physically present in clinics to reduce delays between the study offer by the clinician and researcher contact. Written study information and weekly email reminders were appreciated alongside prompt responses to clinical queries. The music therapists reported challenges in not assessing participants prior to groups and suggested that group allocations post-intervention should take into account individual characteristics beyond capacity to attend a morning or afternoon group. There were further challenges as the music therapists worked across more than one clinical borough, requiring rapid familiarisation with wider clinical teams. Similarly, where participants did not clearly fall under a specific care pathway, this led in some cases to the music therapists having to case hold whilst awaiting allocation to the relevant team. Music therapists reported joint working with the research team as supportive especially when linking up for weekly process measures which often provided further evidence to back up clinical concerns.

Participants spoke positively about their experiences of participating in research even if their experience in music therapy was less so. Some likened being invited to “winning the lottery”. Written materials were helpful as were consistent and clear communication. Whilst waiting for the allocation caused some apprehension, participants felt well-enough informed to accept that this was something they had signed up to. Participants valued the relationships that they built with researchers and the continuity of seeing the same person each time along with flexibility for appointments. They cited understanding, friendliness, support, encouragement to attend the next appointment and being thanked for their time as important. The vouchers provided after assessments were welcomed and cited as a good incentive to continue with research assessments. One participant suggested smaller denominations so that there was more flexibility in what could be purchased.

#### Acceptability of outcome measures

Outcome measures were generally acceptable to participants with < 1% of items missing. No items were missing on the primary measure of the MADRS. Three participants struggled to answer CSQ questions relating to services before they attended music therapy (e.g. CSQ-B – Did you get the kind of service you wanted?). A few participants declined to answer questions relating to sex (MANSA item 13, BDI-II item 21). Items 17, 18 (taking and accepting medication) and 25 (problems living with others) of the LSP were most often rated as not applicable by researchers.

Some participants found the assessment questions anxiety provoking but the majority stated they found them helpful and appreciated that they went into depth about current issues and provoked reflection on how things were right now. The length of follow-up duration was also appreciated. Participants who were less literate suggested that it was challenging to complete but that researchers gave sufficient support in order to answer the questions. The most problematic assessment was the LSP, which researchers found awkward to administer in a face-to-face interview. Introductory text was added to explain the purpose of the questions to facilitate this. The CSRI also required updating when participants noted that the benefits system had changed to those that were in the questionnaire. Participants particularly appreciated the process measures which they stated helped them to notice changes from week to week.

### Feasibility and acceptability of the intervention (objective 6)

#### Compliance

**Fig. 3:**
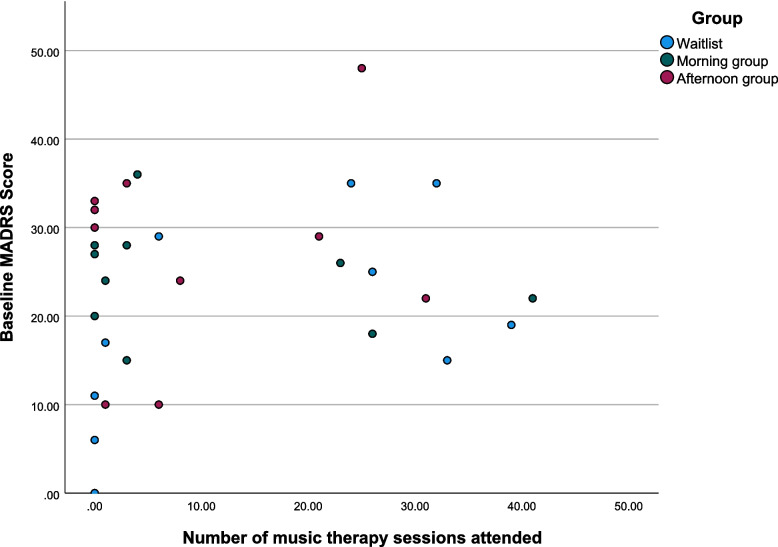
Scatter plot of baseline MADRS score and number of music therapy sessions attended by group

Mean attendance was 10.5 (SD 13.8) out of a possible 42 sessions (25%) with modes of 3 group members per session in one group and 2 group members per session in the other. Participants split into compliant (*N* = 6, mean 27.8/66% sessions), non-compliant (*n* = 8, mean 3.5/8% sessions) and non-attenders (*n* = 6). Five out of six compliant attenders had lower MADRS scores than non-compliant, although one compliant attender scored the maximum (range 18–48) (Fig. 3).

Reasons for non-attendance linked directly to study withdrawal. Four participants with low baseline MADRS scores (< 15) withdrew early on. One wait-list participant who was recruited from a CMHT scored 0 on the MADRS and withdrew prior to the 1 week post-intervention follow-up. Two were participants recruited from Talking Therapies who both withdrew due to commencing employment (one having attended 3 sessions). One participant recruited from the CMHT withdrew due to childcare issues having attended one session.

Two out of the four participants recruited from GP practices did not attend despite scores of > 30 on the MADRS, one due to housing and carer issues and one due to loss of contact. The remaining four non-attending participants had baseline MADRS scores ranging from 20 to 30 and did not attend due to venue accessibility, worsening of symptoms and being unable to commit to the group and life events.

Of the eight non-compliant attenders, one was withdrawn to risk, two requested to withdraw from the group due to group conflict and one left due to commencing employment. The remaining four attended over the course of therapy but faced significant challenges due to refugee status, carer responsibilities, homelessness and family illness.

Based on low attendance figures, we opened up places to non-study participants for the wait-list group. Two additional patients were offered places left by the two study withdrawals but did not complete any study assessments or measures. One attended regularly and one did not attend due to worsening of symptoms prior to the group starting. Of the wait-list study participants, attendance was higher (mean 19.4/46%, SD 15.8) with mode of 5 participants per session. Five participants were compliant (mean 30.8/73% sessions). One ceased attendance after a single session and lost contact with the research team, one after 6 sessions and one did not attend.

#### Adherence

Mean manual adherence was 44.45% (SD 25.94) with moderate reliability when coded by an independent rater. The music therapists used all components of the manual over the course of the groups but with different sections being used at particular times in the therapy process (for example, greater focus on introductory activities in early sessions, recording happening later on in the therapy process). In the two treatment groups, seven song recordings were made. One instrumental recording and a number of improvisations were made in the wait-list group.

The music therapists suggested that further instruction on how to complete adherence forms would have built their confidence alongside a different design of the forms that allowed for a less linear approach to the group process.

#### Process measures

**Fig. 4:**
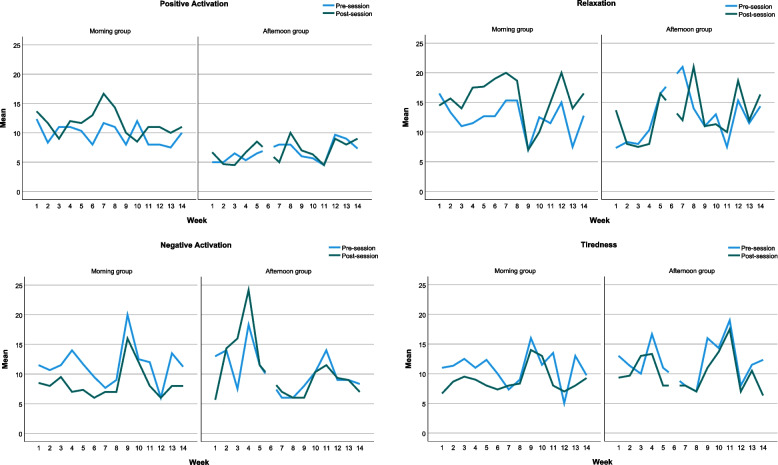
4-Dimensional mood and subscales pre- and post-session, plotted by week and group

**Fig. 5:**
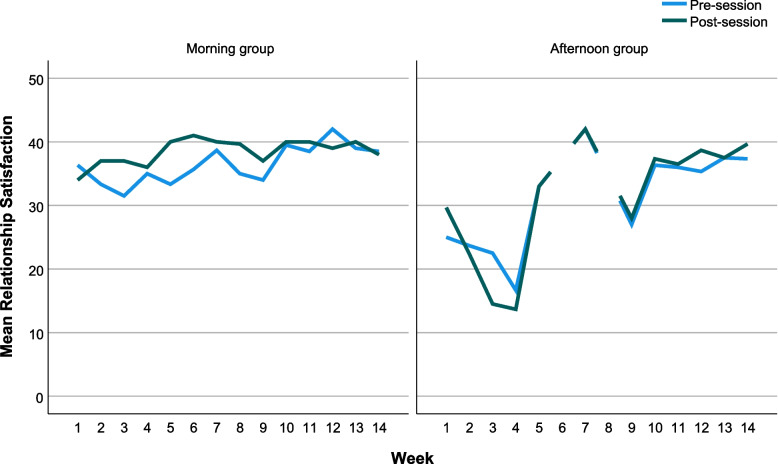
Relationship satisfaction scores pre- and post-session, plotted by week and group

**Fig. 6:**
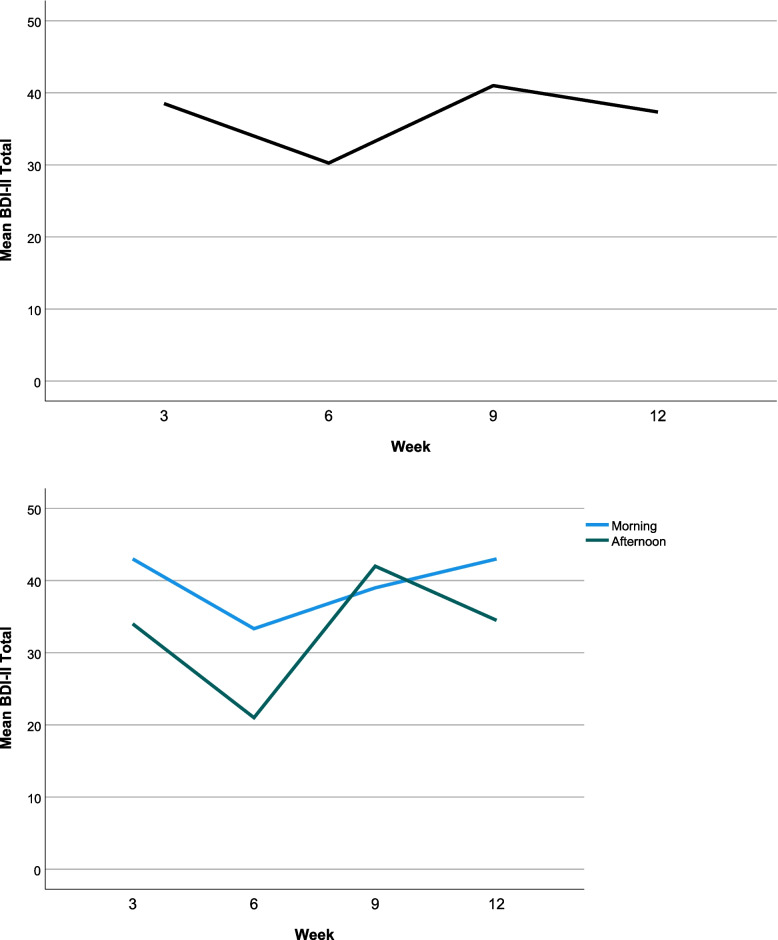
Depression scores on the BDI-II for whole sample and by group in weeks 3, 6, 9 and 12

Due to low attendance, process measures of mood and relationship satisfaction were available for only ten participants (morning group: 6/10, afternoon group: 4/10) and only six for depression (BDI-II: morning group 3/10, afternoon group 3/10). Plots of pre and post mood scores (Fig. 4) suggested an increase in positive energy, relaxation and reduction in tiredness and negative activation in the morning group alongside improvements in relationship satisfaction (Fig. 5). The afternoon group demonstrated a different picture whereby earlier sessions reported an increase in negative activation and lower relationship satisfaction scores in the first 4 weeks and less marked mood differences pre- and post-session. For the BDI-II (Fig. 6), depression scores reduced in both groups between weeks 3 and 6, but then increased again between weeks 6 and 9. There was a reduction in depression in week 12 in the afternoon group.

#### Experiences of the intervention

Ten participants took part in qualitative interviews. In terms of group experiences, three superordinate themes were identified: The group as a happy and safe place; Music stimulates new feelings and songwriting aids expression into words; Uncertainty, unmet needs and the ending were challenging. Further detail on these experiences of the therapy can be found in Windle et al. [85]. Participants reported an average of 5 changes (range 1–9) whilst referring clinicians reported observing an average of three changes in their patients. The majority of these changes were positive, the most common being linked to musical engagement, changes in mood and confidence. Three participants reported increased engagement in other activities whilst three reported negative changes in terms of nervousness, feeling worse at the end of therapy and becoming more housebound. Three of the wait-list group participants reported changes they had hoped for, but did not happen, namely: a wish to change memory of trauma, to change how they thought and a wish to have been more involved in the group. Participants tended to be surprised by the changes that they had noticed (65% of changes were rated as 4 or 5 on the Client Change Interview expectancy-surprised scale) and believed them to be unlikely to have happened without therapy (58% of changes rated as 1 or 2 on the likelihood scale). All participants rated their changes as moderately to extremely important (3–5 on the importance scale).

Accessibility of the therapy location, session frequency and managing the group ending were described as challenging by participants. Participants suggested longer sessions (e.g. 2 hours) but twice per week would be preferable to three times per week.

The music therapists reported challenges in the make-up of each therapy group alongside high levels of drop-out and the impact on group members. Further attention to the make-up of the group was suggested post-randomisation to ensure a good mix and balance of participants.

The music therapists spoke positively about the potential of group songwriting for this client group, especially techniques of song-sharing and combining check-ins and improvisation as a basis for songwriting. They reported some challenges in group songwriting that were beyond their usual scope of practice. Deciding how far to intervene in the songwriting process was described as challenging in the beginning but they observed greater sophistication in the groups’ ability to create over time. Technology, whilst opening up new musical and recording possibilities was a challenge and they suggested that the manual should include more on editing and recording processes.

#### Potential harms and unintended effects

A total of six adverse events (four in the treatment arm, two in the control) and one serious adverse event (treatment arm) were reported during the study (Table 7) in seven different participants. All but one (fainting during a research assessment) were expected events.

**Table 7:** Adverse events and classification by treatment arm

Event	Classification	During treatment	During follow-up assessments	Treatment *N* = 20	Control *N* = 10	Expected?	Related?
Verbal threat	Adverse event	1	0	1	0	Expected	Probably unrelated
Increased suicide risk	Adverse event	1^a^	2	2	1	Expected	Unrelated
Disclosure of risk to self/others	Adverse event	0	1	1	0	Expected	Probably unrelated
Hospitalisation	Serious adverse event	0	1	1^b^	0	Expected	Unrelated
Faint during research assessment	Adverse event	0	1	0	1	Unexpected	Unrelated
Homelessness	Safeguarding alert	2	0	2	0	Unexpected	Unrelated
Total number of events		4	5	7	2		

^a^Risk identified during research assessment after the therapy group

^b^Participant did not attend any group sessions

The most frequent adverse event was increased suicide risk, identified during the research assessments. One participant disclosed a risk to self/others in a follow-up assessment which appeared unrelated directly to the intervention but could possibly have been related to the recent ending of the group. Within the treatment arm, events that occurred during the treatment phase included one verbal threat and one increased suicide risk, identified during completion of process measures. The verbal threat was assessed as probably unrelated given this participant’s risk history although it is not possible to say for certain if events in the group were a contributing factor. Two instances of homelessness were also reported which, whilst not meeting the definition of an adverse event, were reported as safeguarding alerts following local Trust policies.

Hospitalisation of one treatment arm participant happened during the follow-up assessment period and was reported as a serious adverse event. This participant did not attend any group sessions and withdrew without completing further assessments.

### Health service use (objective 7)

Health service contacts reduced in both groups with a greater reduction in the treatment arm. There were no further hospital admissions for mental health problems in either arm post-baseline. Third sector contacts for self-help and leisure activities increased from baseline in the treatment arm 1 week post-intervention and 6 months follow-up but were reduced at 3 months follow-up.

## Discussion

This feasibility trial piloted a group songwriting music therapy intervention for patients with long-term depression and assessed the feasibility and acceptability of both the intervention and of conducting a larger randomised controlled trial. Descriptive information on health service use was collected to inform a future health economic evaluation.

- Feasibility and acceptability of research methodology

The overall research methodology was feasible and acceptable. Recruitment was most successful in secondary care community mental health teams and via self-referrals from patient and public groups. Success may be due to the research team’s familiarity recruiting in such services or potentially due to a higher threshold of symptom severity held by these services. Our approaches through GP practices were by letter only and it remains to be seen if recruitment could have been more successful if researchers were available during clinic time to speak to those who express interest to their GP. Similarly, there was limited success in recruiting from Talking Therapy services, possibly due to lower symptom thresholds and recent receipt of talking therapy. Instances of unblinding were due to participants contacting researchers post-randomisation. Provision of a different contact telephone number post-randomisation might help to manage communications and maintain blinding.

In terms of clinical outcomes, there were differences between observer and self-reported measures of depression. Whilst participants did not report large changes between assessments, both blinded researchers and clinicians who were interviewed, reported wider observed changes. This may be due to the chronicity of symptoms experienced by participants making it challenging to notice change (for example, the BDI-II asks for changes in the last 2 weeks) [87]. We would therefore propose the MADRS as a suitable measure for the primary outcome of a future trial alongside secondary measures of psychological distress, quality of life and life skills.

Outcomes suggest a promising effect on the reduction of depression and improved social adjustment. However, these improvements were not seen until 3 months post-intervention, suggesting this as the point at which greatest improvement might be seen. Eight treatment participants and four wait-list participants scored worse for their depression symptoms at post-intervention. There are two possible explanations. One is that for treatment participants, the ending of an intense social experience was challenging and therefore measures picked up low mood for treatment participants at this endpoint. Further preparation, signposting and support of participants for their “next steps” might help to ameliorate this. Alternatively, the worsening of symptoms might be attributed to the time of year the measures were taken as this occurred at the post-intervention follow-up which took place towards the end of January [88, 89]. Finally, symptom improvements at the post-intervention follow-up in three wait-list arm participants may also capture their expectancy as they awaited to start their own groups [90], or they might capture spontaneous improvement.

(b) Feasibility and acceptability of intervention

Whilst overall elements of the intervention appeared feasible, a number of areas require modification prior to any further testing. Attendance was poor in treatment groups, but slightly better for the wait-list group. A number of factors may help to explain this: Non-attending participants tended to either have (a) low symptom severity scores (< 15 on the MADRS), (b) were recruited from Talking Therapies and commenced employment or (c) felt there was too much going on to be able to commit to attendance. Childcare, housing and multiple appointment demands were the main reasons cited for being unable to commit. There was also a difference between morning and afternoon groups. Participants were given the option to choose which time they would prefer, and noticeably, those with more severe depression scores chose the later time in the afternoon.

The group frequency of 3 times per week was not feasible for this client group, hindered also for many by the group location. Participants suggested that twice per week would be more manageable in end interviews. Challenges in attendance are known for this patient population [91] and a number of participants faced significant issues with complex life situations including homelessness, care responsibilities and safeguarding. Modifying the session duration and frequency might also mitigate the challenges faced at the end of treatment by participants and potentially improve outcomes at post-intervention. Whilst the intervention included signposting of participants to wider community arts and social opportunities at the end of treatment, few participants attended these final sessions. It may therefore be important to arrange individual follow-up meetings post-intervention to review therapy progress and explore next steps.

Process measures identified important elements of the group culture that may impact upon outcomes. The relationship satisfaction scale in particular gave a good indication of group cohesion and moments of conflict within the group. It may be that greater time was required in one group for the music therapists to foster trust and build a therapeutic relationship [9] prior to commencing the task of writing songs. It is known that early group cohesion is a predictor of later outcomes [92, 93], thus these measures will be useful in explaining outcomes.

The music therapists commented on the lack of control regarding group composition, resulting in groups with large differences in levels of musicianship and also groups where participants were already familiar with each other through other services. Neither of these variables were considered in the trial, yet both critical mass and homogeneity of musical preferences are important factors in therapeutic group songwriting [94, 95]. In a larger randomised controlled trial, it would be challenging to curate group composition post-randomisation as this would rely on sufficient recruitment up-front and may result in long delays between consent and commencement of the intervention. This poses a risk of attrition and potentially long waits for those who have enrolled onto the study as well as resource challenges in delivering a larger number of groups all together, rather than a more staggered approach [96].

This study encountered issues in the music therapists’ use of recording software as part of the intervention. Modifications to the intervention include more support for music therapists on editing and recording songs within sessions and further skills training in the technology. Participants suggested longer sessions of up to 2 hours would be beneficial to allow for these processes. The adherence form also requires re-design to capture adherence to core group principles without relying upon a linear group process.

(iii) Assessment of service use

This was relatively simple to ascertain from participants although further patient and public involvement will be important to ensure benefits and related health economic questions are relevant and up to date.

## Consideration of intervention attendance and study withdrawals

This study had a high number of withdrawals (*N* = 12, 40%), most having occurred by the point of 1 week post-intervention. It was notable that all bar one of the non-attending participants in the treatment arm (*N* = 5) chose to withdraw from the study despite encouragement to continue with assessments. For these participants, elements of housing, caring and life made the thought of further participation too difficult. For the one participant who did not withdraw, contact was lost and the research team were unable to complete any of the follow-up assessments with this person. All other withdrawals were with participants who attended fewer than ten sessions. Further examination of the factors preventing group attendance is therefore important prior to conducting a future trial. Group attendance is known to be a challenge for this patient group [90] and strategies to address this include ensuring full information about the intervention, offering assessment or trial sessions and curating the location and time to be as accessible as possible. Further qualitative exploration with participants for example, regarding barriers such as housing, appointments and childcare, may help to identify exactly how and when group music therapy may be appropriate and accessible. Further stratification of patient characteristics may be useful in a larger trial [97]. For example, stricter eligibility criteria on depression severity (e.g. a cut-off score of 20 on the MADRS) may help to avoid recruiting those with minimal depression scores who attend fewer sessions and it may also help to identify those who will struggle to attend due to a greater severity of symptoms and associated life factors. Recruitment may be most successful from secondary care mental health services and this may also aid retention. Similarly, it will be important to balance randomisation on core characteristics of age, gender, duration of depression and symptom severity.

## Limitations

The study is limited by necessarily small numbers; hence, all outcomes are descriptive only and may not be representative of any true effect. The loss of follow-up data from those participants who withdrew and may not have benefitted from the intervention may similarly have impacted the outcomes reported. However, three out of four participants who withdrew from the intervention due to negative experiences or feeling there was not benefit still took part in assessments and were included in the outcome data. Recruitment was from one NHS site in East London and therefore findings may be limited in their generalisability to other settings.

## Conclusion

Based on the study feasibility criteria, a randomised controlled trial of songwriting in group music therapy is feasible and acceptable but further developments and modifications—especially to the intervention and, also, the trial design—are required.

In terms of study design, recruitment should focus on community mental health teams, and link to patient and public forums. A recruitment rate of 4 patients per week can be expected, but time should be factored in to allow a slower recruitment rate at the start. Inclusion criteria should include screening for depression severity prior to informed consent. Randomisation should stratify for age, gender and duration of depression and include an active control to minimise any expectancy effect of treatment. Outcomes immediately post-intervention may be influenced by the treatment ending with benefits potentially detected at 3 months.

Regarding the intervention, further piloting is required to refine the intervention and to determine the primary endpoint. Further intervention development is required to promote greater attendance and group cohesion. Introductory meetings, group location and transportation should be considered carefully. Groups should be less frequent with a longer course (e.g. 2 per week over 6 months) and require a critical mass of at least 4 members. More time is required to prepare for ending and after-care procedures.

## Supplementary Information

**Additional file 1.**  Music Therapy Song Writing Group Session Intervention Manual. SYNCHRONY music therapy group songwriting intervention manual and logic diagram used in this study.

## Data Availability

The datasets used and analysed for this study are available from the corresponding author on reasonable request.

## Abbreviations

BDI-II: Beck Depression Inventory II
BSI: Brief Symptom Inventory
BSI ANX: BSI Anxiety subscale
BSI DEP: BSI Depression subscale
BSI GSI: BSI Global Severity Index
BSI HOS: BSI Hostility subscale
BSI IIS: BSI Interpersonal Sensitivity subscale
BSI OC: BSI Obsessive–Compulsive subscale
BSI PAR: BSI Paranoia subscale
BSI PHOB: BSI Phobia subscale
BSI PSDI: BSI Positive Symptom Distress Index
BSI PST: BSI Positive Symptom Total
BSI PSY: BSI Psychoticism subscale
BSI SOM: BSI Somatisation subscale
CI: Confidence interval
CBASP: Cognitive behavioural analysis system of psychotherapy
CBT: Cognitive behavioural therapy
CD: Compact disc
CMHT: Community Mental Health Team
CONSORT: Consolidated Standards of Reporting Trials
CSQ: Client satisfaction questionnaire
CSRI: Client services receipt inventory
DMS: Dispositional Mood Scale
EDGAR-II: Experimental Design Generator and Randomiser
GP: General Practice
GPSES: General Perceived Self-efficacy Scale
HCPC: Health and Care Professions Council
IAPT: Improving Access to Psychological Therapies
ICD10: International Classification of Diseases and Related Health Problems (version 10)
IPT: Interpersonal Psychotherapy
LSP: Life Skills Profile
LSP CAR: LSP Self-care subscale
LSP COM: LSP Communication subscale
LSP NON: LSP Non-turbulence subscale
LSP RESP: LSP Responsibility subscale
LSP SOC: LSP Social Contact subscale
LSP SUM: LSP Sum score
MANSA: Manchester Short Quality of Life scale
MADRS: Montgomery-Åsberg Depression Rating Scale
MCID: Minimal clinically important difference
NHS: National Health Service
RSES: Rosenberg self-esteem scale
RSS: Relationship Satisfaction Scale
SD: Standard deviation
UK: United Kingdom
WSAS: Work and social adjustment scale

## References

[1] World Health Organisation. The world health report 2001 – Mental health: new understanding, new hope. World Health Organisation. 2001. https://apps.who.int/iris/bitstream/handle/10665/42390/WHR_2001.pdf?sequence=1&isAllowed=y. Accessed 06 Jul 2022.

[2] Singleton N, Bumpstead R, O’Brien M, Lee A, Meltzer H. Psychiatric morbidity among adults living in private households 2000. Office for National Statistics. 2001. https://webarchive.nationalarchives.gov.uk/ukgwa/20160105160709/http://www.ons.gov.uk/ons/rel/psychiatric-morbidity/psychiatric-morbidity-among-adults-living-in-private-households/2000/psychiatric-morbidity-among-adults-living-in-private-households.pdf. Accessed 06 Jul 2022. 10.1080/0954026021000045967 12745312

[3] Hölzel L, Härter M, Rees C, Kriston L (2011). Risk factors for chronic depression – a systematic review. J Affect Disord.

[4] Koekkoek B, van Meijel B, Hutschemaekers G (2008). Clinical problems in the long-term care of patients with chronic depression. J Adv Nurs.

[5] Gilmer WS, Trivedi MH, Rush AJ, Wisniewski SR, Luther J, Howland RH (2005). Factors associated with chronic depressive episodes a preliminary report from the STAR-D project. Acta Psychiatr Scand.

[6] HM Government (2001). Safety first: Five-year report of the national confidential Inquiry into suicide and homicide by people with mental illness. London: Department of Health publications. https://documents.manchester.ac.uk/display.aspx?DocID=37603.Accessed 06 Jul 2022.

[7] Berndt ET, Koran LM, Finkelstein SN, Gelenberg AJ, Kornstein SG, Miller IW (2000). Lost human capital from early-onset chronic depression. Am J Psychiatry.

[8] Brown GW, Craig TK, Harris TO (2008). Parental maltreatment and proximal risk factors using the Childhood Experience of Care and Abuse (CECA) instrument: a life-course study of adult chronic depression. J Affect Disord.

[9] Jobst A, Brakemeier E-L, Buchheim A, Caspar F, Cuijpers P, Ebmeier KP (2016). European psychiatric association guidance on psychotherapy in chronic depression across Europe. Eur Psychiatry.

[10] Dougherty LR, Klein DN, Davila J (2004). A growth curve analysis of the course of dysthymic disorder: the effects of chronic stress and moderation by adverse parent-child relationships and family history. J Consult Clin Psychol.

[11] Durbin CE, Klein DN, Schwartz JE (2000). Predicting the 2 ½-year outcome of dysthymic disorder: the roles of childhood adversity and family history of psychopathology. J Consult Clin Psychol.

[12] Klein DN, Arnow BA, Barkin JL, Dowling F, Kocsis JH, Leon AC (2009). Early adversity in chronic depression: clinical correlates and response to pharmacotherapy. Depress Anxiety.

[13] Klein DN, Santiago NJ (2003). Dysthymia and chronic depression: introduction, classification, risk factors and course. J Clin Psychol.

[14] Wiersma JE, Hovens JG, van Oppen P, Giltay EJ, van Schaik DJ, Beekman AT (2009). The importance of childhood trauma and childhood life events for chronicity of depression in adults. J Clin Psychiatry.

[15] Teicher MH, Samson JA (2013). Childhood maltreatment and psychopathology: a case for ecophenotypic variants as clinically and neurobiologically distinct subtypes. Am J Psychiatry.

[16] Angst J, Gamma A, Rossler W, Ajdacic V, Klein DN (2011). Childhood adversity and chronicity of mood disorders. Eur Arch Psychiatry Clin Neurosci.

[17] Chu DA, Williams LM, Harris AW, Bryant RA, Gatt JM (2013). Early life trauma predicts self-reported levels of depressive and anxiety symptoms in nonclinical community adults: relative contributions of early life stressor types and adult trauma exposure. J Psychiatr Res.

[18] Lizardi H, Klein DN, Ouimette PC, Riso LP, Anderson RL, Donaldson SK (1995). Reports of the childhood home environment in early-onset dysthymia and episodic major depression. J Abnorm Psychol.

[19] Uher J (2014). Persistent depressive disorder, dysthymia and chronic depression: update on diagnosis, treatment. Psychiatric Times.

[20] Ruhe HG, van Rooijen G, Spijker J, Peeters FPML, Schene AH (2012). Staging methods for treatment resistant depression a systematic review. J Affect Disord.

[21] Kocsis JH (2003). Pharmacotherapy for chronic depression. J Clin Psychol.

[22] Silva de Lima, M, Moncrieff J, Soares B. Drugs versus placebo for dysthymia. Cochrane Database Sys Rev. 2005. 10.1002/14651858.CD001130. 10.1002/14651858.CD001130 11034701

[23] Michalak EE, Lam RW (2002). Breaking the myths: new treatment approaches for chronic depression. Can J Psychiatry.

[24] Cuijpers P, Noma H, Karyotaki E, Vinkers CH, Cipriani A, Furukawa TA (2020). A network meta-analysis of the effects of psychotherapies, pharmacotherapies and their combination in the treatment of adult depression. World Psychiatry.

[25] Cuijpers P, van Straten A, Schuurmans J, van Oppen P, Hollon DS, Andersson G (2010). Psychotherapy for chronic major depression and dysthymia: a meta-analysis. Clin Psychol Rev.

[26] Wolff AV, Hӧlzel LP, Westphal A, Härter M, Kriston L (2012). Combination of pharmacotherapy and psychotherapy in the treatment of chronic depression: a systematic review and meta-analysis. BMC Psychiatry.

[27] Van Bronswijk S, Moopen N, Beijers L, Ruhe HG, Peters F (2018). Effectiveness of psychotherapy for treatment-resistant depression: a meta-analysis and meta-regression. Psychol Med.

[28] Fonagy P, Rost F, Carlyle JA, McPherson S, Thomas R, Pasco Fearon RM (2015). Pragmatic randomized controlled trial of long-term psychoanalytic psychotherapy for treatment-resistant depression: the Tavistock Adult Depression Study (TADS). World Psychiatry.

[29] Visentini C, Cassidy M, Bird VJ, Priebe S (2018). Social networks of patients with chronic depression: a systematic review. J Affect Dis.

[30] Trondalen G (2017). Relational music therapy: an intersubjective perspective.

[31] Aalbers S, Fusar-Poli L, Freeman RE, Spreen M, Ket JCF, Vink AC (2017). Music therapy for depression. Cochrane Database Syst Rev.

[32] Gold C, Mӧssler K, Grocke D, Heldal TO, Tjemsland L, Aarre T (2013). Individual music therapy for mental health care clients with low therapy motivation: multicentre randomised controlled trial. Psychoth Psychosom.

[33] Grocke D, Bloch S, Castle D, Thompson G, Newton R, Stewart S (2013). Group music therapy for severe mental illness: a randomized embedded experimental mixed methods study. Acta Psychiatr Scand.

[34] Chanda ML, Levitin DJ (2013). The neurochemistry of music. Trends Cogn Sci.

[35] Kreutz G (2014). Singing and social bonding - introduction. Music Med.

[36] Rolvsjord R (2010). Resource oriented music therapy in mental health care.

[37] Slade M (2009). Personal recovery and mental illness.

[38] Solli HP, Rolvsjord R, Borg M (2013). Toward understanding music therapy as a recovery-oriented practice within mental health care: a meta-synthesis of service users’ experiences. J Music Ther.

[39] Stern D (2010). Forms of vitality: Exploring dynamic experience in psychology, the arts, psychotherapy and development.

[40] Erkkilä J, Ala-Ruona E, Punkanen M, Fachner J, Hargreaves D, Miell D, Macdonald R (2012). Creativity in improvisational psychodynamic music therapy. Musical imaginations: multidisciplinary perspectives on creativity, performance and perception.

[41] Erkkilä J, Punkanen M, Fachner J, Ala-Ruona E, Pöntiö I, Tervaniemi M, Vanhala M, Gold C (2011). Individual music therapy for depression: randomised controlled trial. Br J Psychiatry.

[42] Baker F, Wigram T (2005). Songwriting: Methods, techniques and clinical applications for music therapy clinicians, educators and students.

[43] Gold C, Solli HP, Krüger V, Lie SA (2009). Dose-response relationship in music therapy for people with serious mental disorders: systematic review and meta-analysis. Clin Psychol Rev.

[44] Rolvsjord R, Gold C, Stige B (2005). Research rigour and therapeutic flexibility: rationale for a therapy manual developed for a randomized controlled trial. Nordic J Music Ther.

[45] Thabane L, Ma J, Chu R, Cheng J, Ismaila A, Rios LP (2010). A tutorial on pilot studies: the what, why and how. BMC Med Res Method.

[46] Carr CE, O’Kelly J, Sandford S, Priebe S (2017). Feasibility and acceptability of group music therapy vs wait-list control for treatment of patients with long-term depression (the SYNCHRONY trial): study protocol for a randomised controlled trial. Trials.

[47] Hoffmann T, Glasziou P, Boutron I, Milne R, Perera R, Moher D (2014). Better reporting of interventions: template for intervention description and replication (TIDieR) checklist and guide. BMJ.

[48] Apple. Garage Band for macOS. Version 10.0.3. Cupertino, CA: Apple Inc; 2014.

[49] Eldridge SM, Chan CL, Campbell MJ, Bond CM, Hopewell S, Thabane L (2016). CONSORT 2010 statement: extension to randomised pilot and feasibility trials. BMJ.

[50] Elliott R, Slatick R, Urman M, Frommer J, Rennie DL (2001). Qualitative Change Process Research on Psychotherapy. Qualitative psychotherapy research: Methods and methodology.

[51] Montgomery SA, Åsberg M (1979). A new depression scale designed to be sensitive to change. Br J Psychiatr.

[52] Rush AJ, First MB, Blacker D (2008). Handbook of Psychiatric Measures.

[53] Williams JBW, Kobak KA (2008). Development and reliability of a structured interview guide for the Montgomery-Åsberg depression rating scale (SIGMA). Br J Psychiatr.

[54] Duru G, Fantino B (2008). The clinical relevance of changes in the Montgomery-Asberg depression rating scale using the minimum clinically important difference approach. Curr Med Res Opin.

[55] Hawley CJ, Gale TM, Sivakumaran T (2002). Defining remission by cut off score on the MADRS: selecting the optimal value. J Affect Disord.

[56] Bandelow B, Baldwin DS, Dolbert OT, Andersen HF, Stein DJ (2006). What is the threshold for symptomatic response and remission for major depressive disorder, panic disorder, social anxiety disorder, and generalized anxiety disorder?. J Clin Psychiatry.

[57] Masson SC, Tejani AM (2013). Minimum clinically important differences identified for commonly used depression rating scales. J Clin Epidemiol.

[58] Beck AT, Steer RA, Brown GK (1996). Manual for the Beck Depression Inventory-II.

[59] Dworkin RH, Turk DC, Wyrwich KW, Beaton D, Cleeland CS, Farrar JT (2008). Interpreting the clinical importance of treatment outcomes in chronic pain clinical trials: IMMPACT recommendations. J Pain.

[60] Hiroe T, Kojima M, Yamamoto I, Nojima S, Kinoshita Y, Hashimoto N (2005). Gradations of clinical severity and sensitivity to change assessed with the Beck Depression Inventory-II in Japanese patients with depression. Psychiatry Res.

[61] Wilson HD (2008). Minimum clinical important differences of health outcomes in a chronic pain population: are they predictive of poor outcomes?. Dissertation Abstr Int Section Sci Eng.

[62] Buttons KS, Kounali D, Thomas L, Wiles NH, Peters TJ, Welton NJ (2015). Minimal clinically important difference on the Beck Depression Inventory – II according to the patient’s perspective. Psychol Med.

[63] Derogatis L, Melisaratos N (1983). The brief symptom inventory: an introductory report. Psychol Med.

[64] Ryan C (2007). British outpatient norms for the Brief Symptom Inventory. Psychol Psychother.

[65] Rosenberg M (1965). Society and the adolescent self-image.

[66] Baranik LE, Meade AW, Lakey CE, Lance CE, Hu C, Hua W (2008). Examining the differential item functioning of the Rosenberg Self-Esteem scale across eight countries. J Applied Social Psychol.

[67] Gray-Little B, Williams VSL, Hancock TD (1997). An item response theory analysis of the Rosenberg Self-Esteem scale. Pers Soc Psychol Bull.

[68] Schwarzer R, Jerusalem M, Weinman J, Wright S, Johnston M (1995). Generalized self-efficacy scale. Measures in health psychology: a user’s portfolio.

[69] Atkisson C, Greenfield TK, Sederer LL, Dickey B (1996). The client satisfaction questionnaire (CSQ) scales and the service satisfaction scale-30 (SSS-30). Outcome assessment in clinical practice.

[70] Mundt JC, Marks IM, Shear K, Greist JH (2002). The work and social adjustment scale: a simple measure of impairment in function. Br J Psychiatry.

[71] Priebe S, Huxley S, Knight S, Evans S (1999). Application and results of the Manchester Short Assessment of Quality of Life (MANSA). Int J Soc Psychiatry.

[72] Parker G, Rosen A, Emdur N, Hadzi-Pavlov D (1991). The life skills profile: psychometric properties of a measure assessing function and disability in schizophrenia. Acta Psychiatr Scand.

[73] Hambridge JA, Rosen A (1994). Assertive community treatment for the seriously mentally-ill in suburban sydney - a program description and evaluation. Aust N A J Psychiatry.

[74] Beecham JK, Knapp MRJ, Thornicroft G, Brewin C, Wing JK (1992). Costing psychiatric interventions. Measuring mental health needs.

[75] Huelsman TJ, Nemanick RC, Munz DC (1998). Scales to measure four dimensions of dispositional mood: positive energy, tiredness, negative activation and relaxation. Educ Psychol Meas.

[76] Huelsman TJ, Furr RM, Nemanick JC (2003). Measurement of dispositional affect: construct validity and convergence with a circumplex model of affect. Educ Psychol Meas.

[77] Burns DD (1993). Ten days to self-esteem.

[78] Lancaster GA, Dodd S, Williamson PR (2004). Design and analysis of pilot studies: recommendations for good practice. J Eval Clin Pract.

[79] Sim J, Lewis M (2012). The size of a pilot study for a clinical trial should be calculated in relation to considerations of precision and efficiency. J Clin Epidemiol.

[80] Julious SA (2005). Sample size of 12 per group rule of thumb for a pilot study. Pharm Stat.

[81] Harris T, Brown GW, Robinson R (1999). Befriending as an intervention for chronic depression among women in an inner city. 1 Randomised controlled trial. Br J Psychiatry.

[82] Kocsis JH, Gelenberg AJ, Rothbaum BO, Klein DN, Trivedi MH, Manber R (2009). Cognitive behavioural analysis system of psychotherapy and brief supportive psychotherapy for augmentation of antidepressant nonresponse in chronic depression The REVAMP trial. Arch Gen Psychiatry.

[83] Simpson S, Corney R, Fitzgerald P, Beecham J (2000). A randomised controlled trial to evaluate the effectiveness and cost-effectiveness of counselling patients with chronic depression. Health Technol Assess.

[84] Brown J. Experimental Design Generator and Randomiser (EDGAR-II). 2005. http://www.edgarweb.org.uk/. Accessed 05 Jul 2022.

[85] Windle E, Hickling LM, Jayacodi S, Carr C (2020). The experiences of patients in the synchrony group music therapy trial for long-term depression. Arts Psychother.

[86] Braun V, Clarke V (2006). Using thematic analysis in psychology. Qual Res Psychol.

[87] Kjægaard M, Arfwedson Wang CE, Waterloo K, Jorde R (2014). A study of the psychometric properties of the Beck Depression Inventory-II, the Montgomery and Åsberg depression rating scale, and the hospital anxiety and depression scale in a sample from a health population. Scand J Psychol.

[88] Øverland S, Woicik W, Sikora L, Whittaker K, Heli H, Skjelkvåle FS (2020). Seasonality and symptoms of depression: A systematic review of the literature. Epidemiol Psychiatr Sci.

[89] Harmatz MG, Well AD, Overtree CE, Kawamura KY, Rosal M, Ockene IS (2000). Seasonal variation of depression and other moods: a longitudinal approach. J Biol Rhythms.

[90] Bradt J (2012). Randomized controlled trials in music therapy: Guidelines for design and implementation. J Music Ther.

[91] Dilgul M, McNamme P, Orfanos S, Carr CE, Priebe S (2018). Why do psychiatric patients attend or not attend treatment groups in the community: a qualitative study. PLoS One.

[92] Orfanos S, Priebe S (2017). Group therapies for schizophrenia; Initial group climate predicts changes in negative symptoms. Psychosis.

[93] Tschuschke V, Dies R (1994). Intensive analysis of therapeutic factors and outcome in long-term inpatient groups. Int J Group Psychother.

[94] Myers-Coffman K, Krater C, Shanine M, Bradt J (2020). Feasibility and acceptability of the resilience songwriting program for adolescent bereavement. Arts Psychother.

[95] Baker F (2013). Music therapists’ perceptions of the impact of group factors on the therapeutic songwriting process. Music Ther Persp.

[96] Biggs K, Hind D, Gossage-Worrall R, Sprange K, White D, Wright J (2020). Challenges in the design, planning and implementation of trials evaluating group interventions. Trials.

[97] Saunder R, Cohen ZD, Ambler G, DeRubeis RJ, Wiles N, Kessler D (2021). A patient stratification approach to identifying the likelihood of continued chronic depression and relapse following treatment for depression. J Pers Med.

