# Supplementary material for: Group music therapy with songwriting for adult patients with long-term depression (SYNCHRONY study): a feasibility and acceptability study of the intervention and parallel randomised controlled trial design with wait-list control and nested process evaluation
Source: Pilot Feasibility Stud. 2023 May 5;9:75. doi: 10.1186/s40814-023-01285-3 (PMC10161457; doi:10.1186/s40814-023-01285-3)
Supplement: Supplementary file 1 — Additional file 1. Music Therapy Song Writing Group Session Intervention Manual. SYNCHRONY music therapy group songwriting intervention manual and logic diagram used in this study. [file 40814_2023_1285_MOESM1_ESM.pdf]

Music Therapy Song Writing Group Session Intervention Manual

Requirements

- Maximum number participants : 10
- Room: to seat up to 12 (10 participants, therapist and co-therapist)
- Ideally décor such as paintings, plants, natural light to provide creative environment
- Wifi
- Reasonable level of soundproofing from interior to exterior and vice versa
- Room to be free from interruption or loud external noise for duration of session (90min)
- Group to be run by two music therapists. In the event of 1 therapist being unable to attend (eg. Sickness or emergency leave), cover will be provided by another Trust music therapist. In the event of both therapists being unable to attend, the group will be cancelled.
- Clinical Studies Officer will assist in setting up video camera for research recording, sending reminders to members who request this and receiving any calls from participants running late or cancelling session. CSO will assist with process assessments.

Safety

- Therapist to arrange a number to call when session starts, and when last participant leaves
- Room must have mobile phone signal and/or landline
- Therapist and assistant should ensure clear access to the nearest exit all times.

Session Length

90 minutes, consisting of 60 minutes session with 15 minutes pre-/post- for socialisation

Tips to maximise attendance

- Text/email reminder to all members prior to each session – will be done by Clinical Studies Officer assisting with group. Group members will be asked if this is something they would like to happen.
- Set up a buddy system of travel to groups

Equipment

- Range of large and hand held percussion instruments eg. large: Djembe drum, bongos, conga, snare, tom toms small: cabassa, castanet, cowbell, triangle, various shakers, chimes (hand held or on stand)
- Tuned instruments: guitar, electric keyboard and/or acoustic piano, auto harp, xylophone, ballaphone, marimba, glockenspiel, harmonica, thumb piano, chime bars, hand bells etc.
- Recording equipment: zoom digital recorder, ipad with compatible external microphone and Garageband or similar
- Amplification for ipad and electric guitar/keyboard where required
- Projector to connect to Ipad for song ideas
- Speakers for playback
- Flip chart and blu-tack
- Video camera to record the session.

An induction session will be offered to intervention participants for therapists to assess fit of group membership in the two subsequent groups.

- Therapists introduce themselves, set expectations around what will happen and answer questions. Introduce the equipment and the sorts of music-making that will happen.

Introductions (10 min, 20 min for initial session):

- Therapists to group, group members to each other
- Basic housekeeping- session length, fire exits, facilities etc
- Participants & therapist to introduce themselves with 1-2 sentences about who they are and how they feel about joining the group. Therapists explore pre-existing relationship to music, hopes and expectations. If comfortable, then to introduce themselves and how they are feeling generally musically (instrumentally, or if feeling confident sung improvisation). *NB this instrumental introduction section may be repeated each week as a means of affording everyone a quick 'check in' and allowing therapist to gauge to initial group'climate'*
- After acknowledging introductions and any matters arising, therapist to provide an overview of the planned 14 week schedule:

Session 1 : Introduction to rationale and activities that will be encountered in the group.
Session 2-31 Songwriting and developing group song list
Sessions 32 -49: Review and closure

Ground rules and general principles of the group (first session and where reminders needed)

- Importance of regular attendance
- Participants to try to stay in room for duration of session
- Appropriate language, e.g. no personal comments, swearing, aggression, or homophobic, racist or in any way prejudicial language
- Not to talk over others
- Not to come to the group intoxicated or having used drugs
- Mutual respect, each member has equal rights to make suggestions
- No mobile phones
- Confidentiality, except for any comments indicating risk to self or others
- Any relationships that develop outside of the therapy space to be brought back to the group
- If members wish to bring in their own technology, this is to be used with respect for other members of the group. Therapist clarifies expectations around when technology will and won't be used.
- All musical abilities accepted, no right or wrong contributions, all will be supported to find a form of musical expression that works for them in the group
- Any difficulties or disagreements are a normal part of being in and working as a group – we will try to work through these within the group space, using the music making and verbal reflection. If people need time out, this will be facilitated and you will be supported by the therapist with this.

Song writing: an introduction

Therapist in own words to talk about rationale for song writing in music therapy, touching on:

- Externalising difficult emotions/feelings
- Communicating difficult/ambiguous feelings in a safe 'container'
- Celebrating life / culture / identity
- Creative group activity supporting social relationships, experience of flow
- Self-efficacy
- Developing music and personal resources/confidence

Group structure and format:

Each session will begin with an instrumental/ body warm up and check in and end with a closing reflection. Initial sessions will use reflection on a piece of music brought to the session by a group member, which will then lead into improvisation and then songwriting. The structure of this may become more flexible as sessions progress to tailor to the evolving needs of the group.

- **Instrumental / body warm up (10 min every session)**

Stretching, breathing and vocal warm ups – tailored to energy/confidence level of group and mindful of any physical disabilities, for example:

- Quiet to loud to soft building up around group from hand rubbing to stomping feet & clapping and back
- Stretching high, breathing in, dropping low, making a sound as air expelled

Individual check in:

- Choosing an instrument and sound for 'how I feel right now' – members are not required to say anything, but can offer verbal reflections if they wish.

Various group ice breaker exercises may be used-

- passing a sound/ rhythm round the group, pairing sounds, sharing to build group awareness, listening to and awareness of others.
- taking it in turns to lead a group improvisation supported by others
- instrumental duets/dialogues initiated by eye contact or other nonverbal gesture around the circle
- Structured song with room for individual contributions to get members used to singing as a group.

Main section of each session : 30-50 min

- **Therapist encourage group discussion around 1-2 participants favourite song**

This exercise repeated each week to build up a list of favourite group songs to re-inforce group identity and ownership

Participant(s) to tell group their favourite song, and why. Encouraged to bring their song to the group if they have a recording.

Therapist to encourage participants to discuss subject matter of song, how it relates to them, what they like about song etc – perhaps draw out a key theme / word, such as 'love', 'survival' , 'blue'. Therapist encourages group to think about the music and how this relates to the song's theme. Therapist works to transition from a feeling elicited from this music into a song theme.

If the person bringing the song wishes to (with aid of song books and 911tabs resource on ipad) group may sing the song, led by therapist. Therapist checks in with the person who is bringing the song. This can lead into improvised play/singing.

- **Warm-up group improvisation to prepare for song-writing**

Group takes a theme from the discussion and improvises on this as a group. Therapist supports members to identify an instrument they wish to try or feel drawn to and encourages to try out a selection if their first choice is not what they wanted.

Therapist offers structure where necessary to build group cohesion and responds with usual clinical improvisation techniques (eg. Musical attunement, reflecting, mirroring, holding, containing).

After improvising, the therapists lead a group reflection on the experience of playing, any ideas or themes they would like to take forward into the song.

Any pre-existing musical skills are acknowledged and supported. Therapist works with group to allow skills to be shared and supports group to generate ideas.

- **Song-writing**

If appropriate, the therapists may split into two sub-groups to work on different elements of the song, before coming together to share the ideas generated. The group will focus on the following elements:

a) Creating lyrics

NB: Throughout this and other group discussion activities, therapist and assistant to ensure equal participation of group members where possible, using appropriate verbal and non verbal language to facilitate this

Using flip chart / whiteboard/ PowerPoint projection, therapist to ask participants what they would like to write a song about, what's important to them now – therapist to draw out group themes from discussion

- Initial focus on ideas for chorus/main theme from initial discussion
- Participants may offer single words, whole lines, and chorus topics
- Discussion as to appropriateness of words for chorus/subject focus
- Discussion of role of verse- i.e. often asking questions answered by chorus, building a story arc, contrasting with chorus theme (e.g. hopelessness and hope)

Note : Rhyming may be important but is not essential, as it can support the flow of lyric creation. Simply creating a list of rhyming words relating to a chorus focus may provide as good starting point to generate ideas.

b) Developing the song

The following guideline activities do not necessarily need happen in this order and may be repeated as necessary. When developing the song, the role of the therapist is to maintain a clear link to the overall theme of the song and to explore musical possibilities with the group. The therapist enables and flexibly supports ideas offered by individual group members and where necessary, offers different musical options to be tried out by the group. Over the weeks that therapist holds different reactions to the songs and themes and continues to offer suggestions and possibilities. The therapist strives to ensure at least 2 or 3 musical and lyrical ideas are offered and tried within the group.

The therapists must ensure all participants have an opportunity to contribute and gently support quieter members to offer their ideas and opinions.

- **Choosing genre**
  - How does the chorus/subject focus suit particular genres?
  - Original music or use melody/ subject of existing song as template for the lyrics?
  - Use of participants pre-existing musical skills if appropriate/available. Therapist to offer guidance where necessary on instrumental techniques, or to accompany where appropriate
  - Therapist manages differing musical preferences- ensure balance of contributions so that this is a group song. Any ideas are always brought back to the group.
  - Therapist allows new musical ideas to come into the group.

- **Developing rhythmic structure**
  - Therapist models rhythmic structure, offering alternatives i.e.counting 4/4,3/4, emphasising on beat(common pop/mainstream) or off beat (reggae)
  - Speaking/chanting lyrics may be helpful
  - Experiment with different time signatures, or ipad pre-set rhythms
  - Lyrical structure may need modification to fit rhythm

- **Developing verse/chorus melody**
  - Those confident enough may improvise melodies on voice or on instruments to lyrics
  - Therapist may provide range of improvised melodies, or draw from warm up activities and encourage feedback from participants

- **Choosing mode & harmony**
  - Therapist to model a range of major, minor, pentatonic and other modes as options
  - Therapist to model different harmonic options (eg rich jazz chords, simple pop chords)
  - Group discussion to select appropriate harmonic accompaniment including discussion of contrasting harmonies for verse/chorus if needed
  - Participant pre-existing skills may be drawn upon here
  - Note- developing harmony/chord changes may help generate melody and vice-versa

- **Adding instrumental accompaniment/possibilities for improvisation**
  - Encourage group discussion as to appropriate instruments for accompaniment
  - Participants with particular competence on certain instruments identified earlier in session (or other sessions) by be encouraged to provide input here
  - May offer less confident members role in song performance/ creation particularly those unconfident singing
  - Therapist to encourage peer-to-peer support and ideas for what can be contributed

c) Rehearsing

- Each completed section of song can be rehearsed in weekly run-throughs
- The rehearsal of the song may be recorded to provide a diary and documentation of progress with the song and may be reviewed in the following session.
- Ongoing group discussion as to what an end product might look like. Therapist offers ideas and explores what does it mean to record or perform?
- The format of the end product is explored with options of recording and performance. Therapists explore with the group what to expect from recording process and how participants would like music to be played- i.e. individual preferences (i.e. only for me to hear/for family/care staff to hear) and group preferences (would all like it to be private of want in public domain
- Likely end-points are offering to record in real time in the therapy space or a video of the performance.
- If the group wishes to have external opportunities, to consider MIND and Trust events and to explore how this might be taken forward by the group.

d) End of session

- Check-in to reflect on how I was at the beginning and how I feel right now. Musical check-in and one word.
- Therapist takes the group back to the beginning of the session and encourages reflection on what has happened. One thing that was successful or will stay with them, one thing that was challenging.
- Therapist encourages group feedback and support.
- Reflection on the songwriting process and encouraging group to make decisions as to when a song is finished.
- Therapist highlights interpersonal relationships and interactions- relationships are brought back to the group's music and are supported in the music.

- If the songwriting process has gone well, the group may wish to end on a final rendition of the song (or another in the group's repertoire)

Final sessions

- Final 3 weeks are dedicated to reviewing the songs that were written. This may include rehearsing and recording in a manner that the group have decided will be their end product. The therapists support reflection on the process of writing the song, drawing out significant decisions and interactions, feelings about the song and relationships within the group. The group may wish to write one final song as an overall reflection of their experiences of being in the group.

Scenarios within the group process:

Conflict between members:

The therapists seek as much as possible to bring the conflict back to within the music (e.g. through an improvisation or through modelling of musical ideas). The therapists support thinking about the conflict within the group space, offering opportunities for group reflection and peer support. Therapists may interpret what is happening to the group and reiterate the purpose of the group in terms of developing a means of supporting interpersonal interactions. Therapists may offer an opportunity to use the song-writing process to document and reflect upon the interactions between group members, if the group are open to this.

Non-participation of group member:

The warm-up and improvisational activities are designed to enable participants to quickly engage in music making. Should a group member struggle to participate actively in the music, the therapists will open a reflection within the group of ways in which the person might be supported to participate, acknowledging the challenges in doing so. Structured musical interaction may help to familiarise the group member and build confidence in using instruments.

Adverse reactions to hearing recording of song:

Wider music therapy studies have shown that recording of music requires some care and sensitivity. Participants may be disappointed with how they sound on the recording, or may not wish to be recorded at all.

The therapists will ensure only those happy to be recorded participate in this. Therapists will provide time after listening back to the recording to reflect upon responses to the song. The therapists may offer suggestions based on these responses and encourage group support. Check in with other peoples' responses may provide alternative points of view that can be discussed within the group. There may be the opportunity to revise some of the song ideas which can be taken into the following session.

After the group:

The music therapists follow up immediately with anyone who has had a difficult time or requires further support following the content of the group. Clinicians responsible for the care of the participant are contacted if any new concerns or risks are noted.

Participation and attendance of the group are recorded in the following locations:

- For Care Coordinated Patients, an entry is made on Rio
- The attendance log is completed
- The self-rated adherence log is completed independently by both therapists

Incident and safety reporting:

Any Adverse Events or Serious Adverse Events occurring from the time of written informed consent until 7 days post-cessation of music therapy must be recorded on the Serious Incident form found on the ELFT intranet and require reporting to the Chief Investigator as soon as possible after the occurrence. The Chief Investigator will fax a copy of this form to the Sponsor within 24 hours of becoming aware of the event.

Adverse Events:

An adverse event is any untoward medical occurrence in a subject to whom an intervention has been administered, including occurrences which are not necessarily caused by or related to that intervention. An Adverse Event can therefore be an unfavourable and unintended sign, symptom or disease temporarily associated with study activities.

An adverse event may include:

- A participant exhibiting aggression (nonverbal or verbal behaviour)
- A participant causing harm to another person
- Disclosure of thoughts or plans which may place the individual or others at risk of harm

How to report an adverse event:

If an adverse event either occurs or is identified during the intervention, the music therapist must first contact the Chief Investigator (Catherine Carr). If the event is related to the study, is severe, or results in the intervention being interrupted they are to notify Catherine as soon as the session has ended by telephone. Catherine will then follow up the event with the patient and therapists to record the event on the adverse events log and to establish whether it has been resolved or is continuing. The adverse event will be assessed to establish whether it should be classified as serious, using the guidance below.

If the event is not defined as serious, the Adverse Event will be recorded in the log and case report form of the participant. An assessment will be made by the Chief Investigator liaising with the participant's music therapists and clinican to establish whether it is safe for the participant to continue with the intervention.

Serious Adverse Events:

A serious adverse event may include any untoward occurrence that:

- Results in death
- Is life-threatening
- Requires hospitalisation or prolongation of hospitalisation
- Results in persistent or significant disability or incapacity
- Consists of a congenital anomaly or birth defect or
- Is otherwise considered medically significant by the investigator

This may include:

- A participant making a suicide attempt
- A participant causing life threatening injury to another
- An event occurring during the course of the study which results in hospitalisation or prolongation of existing hospitalisation related to their mental health

Hospitalisation will not be reported if it is for routine treatment, treatment which was elective or pre-planned, hospitalisation for general care where there was no deterioration in condition or treatment on an emergency outpatient basis for an event **not** fulfilling any of the definitions of serious as given above and not resulting in hospital admission.

The Chief Investigator will report all Serious Adverse Events to the Sponsor and Research Ethics Committee if the event was:

- Related to the administration of study procedures
- Unexpected and not listed in the protocol as an expected occurrence.

Inputs	Outputs		Outcomes – Impact		
	Activities	Participation	Short	Medium	Long
Group music therapy 3 times per week	Introduction (session 1 only)	Therapist sets expectations for group, answers questions.	Expectations set for group, facilitating engagement.	Increased commitment and engagement with therapeutic groups.	Building relationships and trust with others.
	Ground rules	Expectations set on group behaviour/boundaries	Therapeutic rapport and safety established.		Engagement with treatment and optimism for recovery.
2 x Music Therapists<br><br>Musical Instruments	Instrumental/body warm up	Therapist offers activities to get participants used to using instruments, voice and body.	Performance anxiety reduced.<br>Increased musical participation.	Identifying, naming and reflecting on emotional state.	Increased ability to manage internal states.
	Individual check-in	Therapist finds out how people feel right now musically or verbally			
Music Technology<br><br>Flipchart and pens	Ice-breaker activities	Therapist offers structured activities to reduce anxiety and get people used to listening to one another.	Increased awareness of others in the group.	Bonding, building of relationships with others in the group.	Widening of social networks.<br>Building of meaningful relationships with others.<br>Improved social functioning.
	Group discussion around favourite song	Therapist takes known song meaningful to participants and reflects on content and music with group. Used to identify initial songwriting theme.	Reduced anxiety.<br>Sharing of self with others.<br><br>Group focus on theme.		Improved work and social adjustment.

**Inputs**	**Outputs**		**Outcomes – Impact**		
	Activities	Participation	Short	Medium	Long
	Warm up group improvisation	Group use improvisation to generate musical ideas. Therapist responds to musical contributions- musical attunement and containment.	Generation of musical ideas. Opportunities to create, explore, learn by experience.<br><br>Promotes musical cohesion, emotional expression.<br><br>Arousal moderated up or down.	Improved mood.<br><br>Building of hope, optimism and coping strategies for the future.<br><br>Building resources to self-manage arousal and negative cognitions of self.	Resources to think creatively and flexibly in wider life.<br><br>Reduced symptoms of depression and anxiety.<br><br>Reduced use of services.<br><br>Improved satisfaction with treatment.
	Song-writing	Therapists lead group in writing a song on a chosen theme offering a range of musical suggestions.	Vocal leading to verbal expression.	Improved ability to communicate inner state.<br><br>Ideas generated on how to promote inner resources (coping, resilience).	Improved self-efficacy and self-esteem.
	Rehearsing	The group consolidate their song-writing by playing back and if appropriate, recording.	Consolidation of effort, immediate end product.	Achievement and bonding with others.	
	End-check in	Therapist checks in with how participants are feeling now.			Improved quality of life.
	Success and challenges	Therapist identifies from each group member one success and one challenge and relates this back to the group, encouraging group support.	Processing of immediate experience.	Increased ability to reflect on inner state and experiences and share with others.	
